# In the line-up: deleted genes associated with DiGeorge/22q11.2 deletion syndrome: are they all suspects?

**DOI:** 10.1186/s11689-019-9267-z

**Published:** 2019-06-07

**Authors:** Zahra Motahari, Sally Ann Moody, Thomas Michael Maynard, Anthony-Samuel LaMantia

**Affiliations:** The Institute for Neuroscience, and Department of Anatomy and Cell Biology, The George Washington University School of Medicine and Health Sciences, Washington DC, 20037 USA

**Keywords:** 22q11DS, Neural development, Cognition, Cardiovascular, Craniofacial, Copy number variants, Polygenic

## Abstract

**Background:**

22q11.2 deletion syndrome (22q11DS), a copy number variation (CNV) disorder, occurs in approximately 1:4000 live births due to a heterozygous microdeletion at position 11.2 (proximal) on the q arm of human chromosome 22 (hChr22) (McDonald-McGinn and Sullivan, Medicine 90:1-18, 2011). This disorder was known as DiGeorge syndrome, Velo-cardio-facial syndrome (VCFS) or conotruncal anomaly face syndrome (CTAF) based upon diagnostic cardiovascular, pharyngeal, and craniofacial anomalies (McDonald-McGinn and Sullivan, Medicine 90:1-18, 2011; Burn et al., J Med Genet 30:822-4, 1993) before this phenotypic spectrum was associated with 22q11.2 CNVs. Subsequently, 22q11.2 deletion emerged as a major genomic lesion associated with vulnerability for several clinically defined behavioral deficits common to a number of neurodevelopmental disorders (Fernandez et al., Principles of Developmental Genetics, 2015; Robin and Shprintzen, J Pediatr 147:90-6, 2005; Schneider et al., Am J Psychiatry 171:627-39, 2014).

**Results:**

The mechanistic relationships between heterozygously deleted 22q11.2 genes and 22q11DS phenotypes are still unknown. We assembled a comprehensive “line-up” of the 36 protein coding loci in the 1.5 Mb minimal critical deleted region on hChr22q11.2, plus 20 protein coding loci in the distal 1.5 Mb that defines the 3 Mb typical 22q11DS deletion. We categorized candidates based upon apparent primary cell biological functions. We analyzed 41 of these genes that encode known proteins to determine whether haploinsufficiency of any single 22q11.2 gene—a one gene to one phenotype correspondence due to heterozygous deletion restricted to that locus—versus complex multigenic interactions can account for single or multiple 22q11DS phenotypes.

**Conclusions:**

Our 22q11.2 functional genomic assessment does not support current theories of single gene haploinsufficiency for one or all 22q11DS phenotypes. Shared molecular functions, convergence on fundamental cell biological processes, and related consequences of individual 22q11.2 genes point to a matrix of multigenic interactions due to diminished 22q11.2 gene dosage. These interactions target fundamental cellular mechanisms essential for development, maturation, or homeostasis at subsets of 22q11DS phenotypic sites.

**Electronic supplementary material:**

The online version of this article (10.1186/s11689-019-9267-z) contains supplementary material, which is available to authorized users.

## Background

**Fig. 1:**
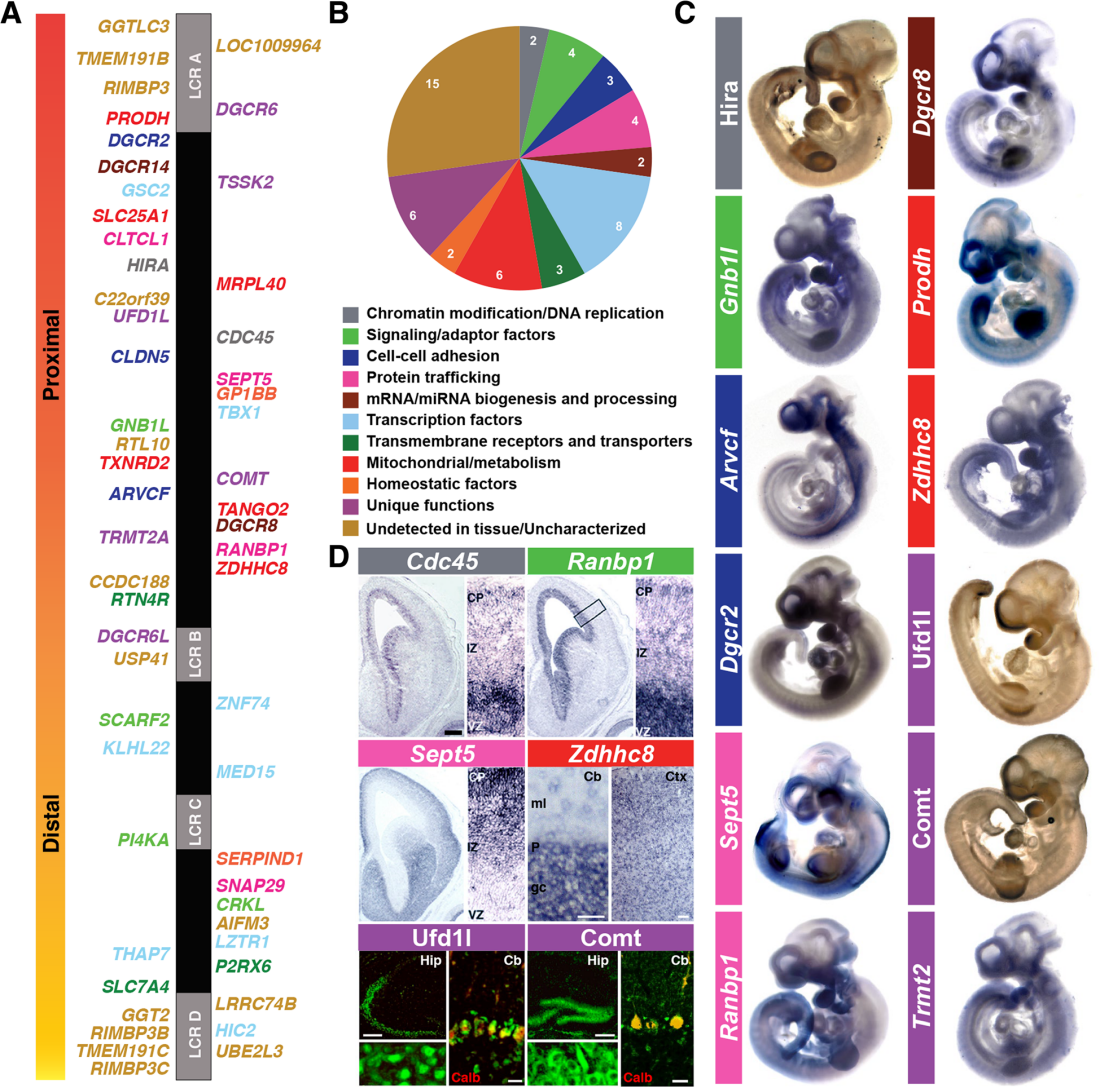
Genes deleted in 22q11DS. **a** Schematic of the 22q11.2 region. Low copy repeats (LCRs) are shown as gray boxes: LCR A-D (not to scale). **b** Protein-coding genes (*n* = 56) are color-coded based on primary, putative, or family member functions as eleven groups. **c** mRNA (*Prodh*, *Zdhhc8*, *Sept5*, *Gnb1l*, *Ranbp1*, *Dgcr8*, *Arvcf*, *Dgcr2*, and *Trmt2a*) or protein (Ufd1l, Hira, Comt) expression of selected genes at mouse embryonic stage E10.5 [58, 73, 169]. **d** Expression localization of *Cdc45*, *Ranbp1*, and *Sept5* in the entire cortical hemisphere of E14.5 embryos (left) and in a higher magnification (right) [60]. Expression pattern of *Zdhhc8* is shown in the adult cerebellum (left) and the cortex (right) [310]. Immunolocalization of Ufd1l and Comt proteins in the hippocampus (left) and cerebellum (right) of the adult mouse brain are shown [58]. *VZ* ventricular zone, *IZ* intermediate zone, *CP* cortical plate, *gc* granular cell layer, *P* purkinje cell layer, *ml* molecular layer, *Cb* cerebellum, *Ctx* cortex. *Hip* hippocampus, *Calb* calbindin. Scale bars: *Cdc45*, *Ranbp1*, *Sept5* = 250 μm, insets = 6.6x, *Zdhhc8* = 50 μm (left) and 100 μm (right). Ufd/Comt: hippocampus (upper left) = 250 μm, insets (lower left) = 10x, cerebellum (right) = 25 μm

The frequency of 22q11.2 deletion—the chromosomal/copy number variant (CNV) that results in most cases of DiGeorge/22q11DS [1, 2]—reflects the unusual genomic architecture of human chromosome 22 (hChr22), position q11.2. At least four repetitive DNA “cassettes” known as low copy-number repeats (LCRs): LCR A, B, C, and D; define the region (Fig. 1a) [6]. These LCRs, due to substantial sequence similarity, facilitate non-allelic homologous meiotic recombination, or “deletion by crossing over of repetitive DNA” [7], resulting in unbalanced translocation, deletions, or duplication [8]. More than 85% of recombination occurs between LCR A and LCR D resulting in a 3 Mb “typical” deletion. The smaller 1.5 Mb “minimal critical” deletion, occurs between LCR A and LCR B in ∼ 10% of affected individuals [6, 8]. Deleted individuals have complex and variable phenotypes including developmental delay, congenital heart defects, craniofacial anomalies such as cleft palate and retrognathia, musculoskeletal anomalies, eye anomalies, hearing loss, absent or small thymus and parathyroid glands, hypocalcemia, compromised immune system, feeding difficulties, and seizures. In addition to these physical manifestations, 22q11.2 deletion syndrome (22q11DS) is associated with behavioral and psychiatric complications such as schizophrenia (SCZ) autistic spectrum disorders (ASD), attention deficit hyperactivity disorder (ADHD), anxiety disorder, as well as intellectual and learning disabilities [3–5]. Clearly, the range of genomic lesions, numbers of genes, and spectrum of phenotypes that define 22q11 CNV disorders defies straightforward explanations of genotype to phenotype correlations. The essential question remains: how CNVs of one, many, or all 22q11.2 genes disrupts developmental and homeostatic mechanisms and complicates the lives of children and adults with 22q11DS.

Individuals with the **A to B** deletion (we use this nomenclature to identify heterozygous elimination of 22q11.2 genes between LCRs A and B) have the full spectrum of phenotypes seen also with the typical **A to D** deletion (i.e., elimination of genes between LCRs A and D), suggesting that key 22q11DS phenotypes are largely due to diminished **A to B** gene dosage [9–11]. Microcephaly and ocular anomalies occur in about 50% of individuals with **A to B** or **A to D** deletions compared to 7% in **B to D** or **C to D** deleted individuals, and cardiovascular defects also appear to be > 3 times more frequent in **A to B** or **A to D** deletions [12]. Nevertheless, deletions in distal regions, **B to D** or **C to D**, have been associated with cardiac developmental anomalies similar to those associated with **A to B** or **A to D** deletions, albeit with lower frequency [13]. Furthermore, the presence and severity of phenotypes among individuals carrying the **A to B** deletion [14] varies significantly, even in siblings who inherit the same deletion from a 22q11.2-deleted parent [15]. In the context of these complex associations between penetrance and severity, it is unlikely that haploinsufficiency of *TBX1* (a 22q11.2 **A to B** gene proposed to explain cardiovascular malformation) [16] or any other single 22q11.2 gene, accounts for the broad range of heart defects in 22q11.2-deleted individuals. This suggests that stochastic modifying interactions between genes in the **A to B** region, as well as unrecognized modifying functions of **B to D** genes, or convergence of subsets of 22q11.2 deleted genes on specific cellular mechanisms play a role in 22q11DS pathogenesis. We will evaluate this question based upon assessment of apparent obligate cellular function (that disrupted by full loss-of-function) of each of the 41 characterized proteins between LCRs A and D, based upon genetic as well as cell biological analyses in human and diverse model systems or organisms.

Phenotypic variability in 22q11DS extends to behavioral diagnoses associated with the syndrome. Several commonly diagnosed disorders, including ASD, are apparently more frequent in **A to B** deleted individuals [12, 17]. Furthermore, several genes from **A to D** including *COMT*, *PRODH*, *GNB1L*, *TBX1*, *SEPT5/GP1BB*, *ZDHHC8*, *PI4KA*, and *ARVCF* have been individually associated with SCZ, ASD, ADHD, and other disorders frequently diagnosed in individuals with 22q11DS [18–20]. Recent population-based exome analysis, however, supports polygenic inheritance of broader, clinically defined behavioral disorders. In ASD, aside from 22q11.2, five additional CNV loci: 1q21.1, 3q29, 7q11.23, 16p11.2, and 15q11.2–13, as well as multiple de novo gene variants have been identified as risk factors [21]. Similarly, SCZ is associated with multiple, rare loss and gain of function mutations in genes that encode calcium channels, postsynaptic cytoskeleton-associated scaffold proteins, and cell adhesion/signaling molecules [22, 23]. Whole exome sequence analyses in individuals with non-syndromic congenital heart defects also fail to support robust individual gene/phenotype correlations [24]. It is thus unlikely that developmental phenotypes in individuals with 22q11.2 CNVs—including commonly diagnosed behavioral deficits associated with neurodevelopmental disorders—arise from the consequences of altered dosage of one single gene within the **A to D** region. Indeed, single gene explanation for individual phenotypes or complete phenotypic spectra are unlikely for most CNV syndromes, including fairly common deletions and duplications at 7q11.23 (Williams Syndrome), 15q11.2 (Prader-Willi Angelman Syndrome), 16p11.2 (Autism Susceptibility), and even whole or partial chromosomal anomalies like trisomy 21 (Down syndrome). Thus, a reassessment of the known protein coding genes at 22q11.2 between LCRs A and D, and their causal relationship to 22q11DS, based upon a thorough review of molecular and cellular mechanisms through which each gene influences development or ongoing physiological function, is necessary, timely, and may have broader resonance for understanding several dosage-related, CNV-associated multigenic neurodevelopmental syndromes.

## Main text

### The 22Q11.2 gene “line-up”: diverse genes on hChr 22q11.2 from A to D

The genes deleted in 22q11DS are conserved across mammals with only modest rearrangements in the human versus mouse minimal deleted regions [3, 25, 26]. Thus, the mouse has emerged as the most common, accessible, genomically valid model for mammalian 22q11.2 gene function [26]. *Drosophila* and *Caenorhabditis elegans* as well as several non-mammalian vertebrates including zebrafish, frog, and chicken also have orthologues of most 22q11.2 genes [3, 25]. There are 154 GenBank entries in the 3 Mb deleted region of hChr22 (LCR A to LCR D; annotation release 108), of which only 56 are predicted to encode proteins: 41 are characterized, while 15 have not been evaluated in any tissue (Additional file 1: Figure. S1). The **A to B** deletion includes 36 (28 characterized) of the 56 apparent protein-coding loci, and the additional 20 (13 characterized) are found in the **B to D** region. The **A to D** interval also contains seven microRNAs, 38 non-coding RNAs, and 53 pseudogenes (Additional file 1: Figure. S1). To better understand the functional and phenotypic consequences of 22q11.2 copy number variation, we divided the 41 **A to D** genes that encode known proteins into ten categories based upon established or inferred primary cellular functions or membership in larger gene families (Fig. 1b). Our criteria included shared functional domains or organelle localization sequences in each of the proteins, common subcellular localization in multiple cell types, and literature reports of apparent obligate functions of each gene. We then re-evaluated available data on specific functions of these protein-coding genes within these categories with a focus on potential contributions to four major 22q11DS phenotypes: heart, face, immune, and brain/behavioral anomalies based upon on the cellular mechanism-based categories.

#### Chromatin modification/DNA replication genes

*HIRA Histone Cell Cycle Regulator* (a.k.a. *DGCR1* or *TUPLE1*; **A to B**) encodes a histone chaperone responsible for replication-independent chromatin incorporation of histone variant H3.3 [27]. HIRA, conserved across eukaryotes, is implicated in transcriptional silencing [28–33]. Its N-terminal domain also functions as a transcriptional activator marking active genes and enhancers [34–37], and potentially involved in transcriptional elongation and recovery after DNA damage [38, 39]. Post-translational modification of HIRA is crucial for *MyoD* activation during muscle cell differentiation [40]. HIRA has two nuclear localization domains and seven WD40 repeats for protein-protein interactions [41, 42]. Among potential interactors, HIRA complexes with Pax3 [43], critical for neural, cardiac, craniofacial, thymic, thyroid, and parathyroid development [44]. In *Xenopus*, *hira* downregulation impairs gastrulation [45]. In mouse and chicken, *Hira* is detected in neural ectoderm, premigratory and migratory neural crest, limb buds, pharyngeal arches, and heart (Fig. 1c) [42, 46]. *HIRA* depletion in chicks increases persistent truncus arteriosus, a key 22q11DS phenotype [47]. Conditional *Hira* deletion in mouse cardiac myocytes alters gene expression, causes pathology and hypertrophy, but does not perturb cardiac morphogenesis [48]. *Hira*^−/−^ mice, however, have severe phenotypes including gastrulation and neurulation defects, growth retardation, and aberrant cardiac development leading to embryonic lethality [41]. No phenotypes have been reported in heterozygous embryos, neonates, or adults.

*CDC45 Cell Division Cycle 45* (**A to B**) is required for initiation, elongation, and coordination of eukaryotic chromosomal DNA replication [49–54]. Data from fission yeast, *Xenopus* egg extracts, and human cells indicate that CDC45 is a limiting factor for replication initiation [55–57]*. Cdc45* is expressed in embryonic brain, pharyngeal arches, thymus and thyroid—where neural crest augments endodermal derivatives (Fig. 1d) [58–60]. *Cdc45*^−/−^ mice have impaired inner cell mass proliferation and die shortly after implantation [61]. Heterozygotes, however, have no apparent phenotypes. Human *CDC45* loss-of-function mutations are linked to craniosynostosis (premature cranial suture closure) and Meier-Gorlin Syndrome, a rare autosomal recessive disorder characterized by growth retardation, microcephaly, small ears and jaws, palatal anomalies, hearing, and vision loss [62], which are very similar to 22q11DS phenotypes [4].

#### Perspective: chromatin modification/DNA replication genes

Apparently, the chromatin remodeling/DNA replication genes *HIRA* and *CDC45*, in the **A to B** region, are necessary for embryonic survival and modulate neural, cardiovascular, and craniofacial development. Loss of function of either gene in mice results in embryonic lethality; however, heterozygotes have no reported anomalies. The appearance of this gene class, chromatin modifiers, in only the **A to B** region elevates their status as likely contributors to key 22q11DS phenotypes. Phenotypic severity, however, may depend upon diminished dosage of other **A to B** genes since neither *Hira* nor *Cdc45* heterozygous deletion in mouse yields detectable phenotypes.

#### Signaling/adaptor factors

*GNB1L G*-*Protein Subunit Beta* (a.k.a. *Wdvcf*; **A to B**) encodes a G-protein/WD40 repeat protein of unknown function [63]. It is highly expressed in mouse forebrain, midbrain, and hindbrain (Fig. 1c) [64]. Homozygous mutation is embryonic lethal, and heterozygotes have pre-pulse inhibition (PPI) deficits, a potentially SCZ-related behavioral phenotype in animal models [64].

*CRKL* C*hicken tumor virus number* 10 *regulator of kinase* (**B to D**) encodes an adapter protein with multiple SH2 and SH3 protein-protein interaction domains [65]. CRK proteins transmit extracellular signals by physically bridging tyrosine-phosphorylated proteins to mediators like Abl tyrosine kinase and guanine-nucleotide exchange factors (GEFs), to influence cell proliferation, differentiation, migration, adhesion, and immune responses [66]. Crkl binds to both phosphorylated Dab1, a mediator of Reelin signaling, and C3G, a Rap1 GEF, to coordinate neuronal migration by cytoskeletal regulation [67–69]. *Crkl* is expressed at high levels in pharyngeal arches, neural crest, and brain, and homozygous deletion compromises neural crest derivatives including cranial ganglia, aortic arch arteries, thymus, parathyroid glands, and craniofacial structures, leading to late-gestation lethality [70]. Homozygous loss-of-function in *Snoopy*, a point *Crkl* mutation [71], also results in craniofacial anomalies, pharyngeal occlusion, and holoprosencephaly. *Snoopy* or *Crkl*^+/−^:*Tbx1*^+/−^ compound heterozygotes (*Tbx1*, an **A to B** gene, see below) have increased retinoic acid (RA) signaling, also seen in mouse embryos with an **A to B** deletion (*LgDel*) [71–74]*.* Crkl may interact with other inductive signaling pathways; fibroblast growth factor (FGF) receptors directly bind to the Ckrl SH2 domain [75]. In 22q11DS, diminished *CRKL* dosage due to **B to D** deletion is associated with impaired T cell and natural killer cell function [76, 77], consistent with CRKL regulation of immune cells [78–80].

*SCARF2 Scavenger Receptor Class F Member 2* (a.k.a. *SREC-II*; **B to D**) encodes a Ca^++^ binding protein that interacts with small molecule ligands such as acetylated low-density lipoprotein (Ac-LDL) [81]. The SCARF2 extracellular domain has multiple epidermal growth factor (EGF)-like repeats and several positively charged residues. Its intracellular domain contains 13 potential phosphorylation sites suggesting a role in cell signaling [81]. *Scarf2* is expressed in neonatal and adult mouse skin, tongue, oral epithelia, and medullary thymus [82]. Its obligate functions in the developing or adult mouse remain unclear; there are no reports of *Scarf2* mutations. *SCARF2* mutations have been linked to Van Den Ende-Gupta syndrome, an extremely rare autosomal recessive disorder characterized by craniofacial anomalies: narrow eye openings, maxillary hypoplasia, flat/wide nasal bridge, everted lower lip, palatal disruptions, prominent ears, skeletal anomalies: scoliosis, long, slender foot and hand bones, mild bowing of long bones, respiratory difficulty due to laryngeal deficits, and cerebellar hyperplasia [83–85]. These overlap with phenotypes seen in 22q11DS [4].

*PI4KA Phosphatidylinositol 4*-*Kinase Alpha* (a.k.a*. PI4KIIIα*; **B to D**) catalyzes synthesis of phosphatidylinositol 4-phosphate (PtdIns4*P*), an essential precursor of PtdIns (4,5) *P*2 and PtdIns (3,4,5) *P3* that mediate receptor-activated phospholipase C (PLC) and phosphoinositide 3-kinase (PI3) signaling [86]. The yeast PI4KA orthologue, *STT4*, is required for cell wall integrity and actin cytoskeleton organization [87]. PI4KA is found in endoplasmic reticulum (ER), nucleoli, and pericentriolar regions, coincident with the Golgi complex [88–90]. Membrane proteins TTC7 and EFR3 recruit PI4KA to the plasma membrane to help maintain protein and lipid composition [91, 92]. In zebrafish, *pi4ka* loss-of-function disrupts brain, heart, and trunk development perhaps due to impaired FGF signaling [93]. PI4KA is expressed in fetal and adult rodent and human brain [94, 95]. Conditional *Pi4ka* inactivation causes lethal gastrointestinal mucosal degeneration, but no obvious neural anomalies [96, 97]. Biallelic *PI4KA* mutation, however, is associated with cerebellar hypoplasia and polymicrogyria [98]. *PI4KA* is also recognized as a SCZ-susceptibility gene in adults with 22q11DS [19, 99].

#### Perspective: signalling/adaptor genes

The **A to B** signaling/adaptor gene, *GNB1L*, may contribute to 22q11DS phenotypes, especially in the brain based upon behavioral evidence; however, current data are inconclusive. The **B to D** signaling/adaptor genes, *CKRL*, *SCARF2*, and *PI4KA*, may modulate craniofacial, cardiac, or cerebral cortical differentiation. Thus, while heterozygous *CRKL*, *SCARF2*, or *PI4KA* deletion may not be necessary for core 22q11DS phenotypes, diminished dosage of one or more of these genes in individuals with the **A to D** deletion may enhance heart, face, or brain phenotypes, yielding more severe impairments or introducing greater phenotypic variation.

#### Cell-cell adhesion genes

*ARVCF Armadillo Repeat Gene Deleted in Velo*-*cardio*-*facial syndrome* (**A to B**) is a member of the Catenin family, which includes α- and β-catenin, plakoglobin, and p120-related proteins [100, 101]. Catenins are critical for adherens junction assembly to facilitate extracellular and intracellular communication. ARVCF protein contains an N-terminus coiled-coil domain, 10 armadillo repeats, and a C-terminal PDZ motif that binds the scaffold protein ERBIN and tight junction proteins ZO-1 and ZO-2 [102–104]. When bound, ARVCF enhances the stability of classical cadherins by reducing their endocytosis [105, 106]. When not bound to cadherin, ARVCF may regulate the cytoskeleton by interacting with small-GTPases such as Rho and Rac [107, 108]. Like most catenins, ARVCF also translocates to the nucleus where it may be involved in alternative mRNA splicing [109, 110]. *ARVCF* is expressed in the human forebrain ganglionic eminences; mouse *Arvcf* is expressed in the brainstem, hippocampus, cerebral cortex, and heart (Fig. 1c) [58, 111]. *Arvcf* depletion in *Xenopus* leads to defects in craniofacial skeleton and aortic arches (also observed in *Tbx1*-deficient tadpoles; see below) [112, 113]. The migration of cranial neural crest is not affected by knocking down of either *arvcf* or *tbx1*; however, their double depletion causes delayed migration suggesting these genes act cooperatively [113]. There are no reported loss-of-function *Arvcf* alleles; however, loss of function of *p120* catenin results in substantially diminished embryonic *Arvcf* expression, in concert with disrupted renal differentiation [114]. *ARVCF* mutations and their related phenotypes do not include structural brain anomalies; however, they have been associated with increased susceptibility to SCZ [115, 116].

*CLDN5 Claudin5*, (a.k.a. *TMVCF*; **A to B**) is a member of the Claudin family, major structural components of tight junctions that are crucial for cell–cell contacts and paracellular permeability, and establishing apical to basolateral intra-membrane borders [117–119]. Cytokines, growth factors, and cadherins regulate claudins [120, 121]. All family members have similar predicted folding and high sequence homology in the first and fourth transmembrane domains and extracellular loops [118], and a C terminus PDZ motif that binds to PDZ peripheral membrane proteins, including ZO-1, -2, and -3, which in turn interact with the actin cytoskeleton to link tight junctions with signal transduction proteins [122, 123]. *Cldn5* is expressed ubiquitously [124, 125]. In *Xenopus*, *cldn5* is strongly expressed in cardiac primordia and its depletion leads to heart malformations [126] and in zebrafish, *cldn5* is required for heart regeneration [127]. *Cldn5*^−/−^ mice, however, do not have cardiovascular malformations. Nevertheless, Cldn5 may modify cardiovascular development via interactions with additional myogenic genes [128, 129]. In the *Dystrophin*^−/−^ (*Dmd1*)/*Utrophin*^−/−^ (*Utrn*) cardiomyopathy model, *Cldn5* mRNA and protein levels decline [129]; in contrast, *Cldn5* overexpression diminishes cardiomyopathy in *Dmd1*^−/−^:*Utrn*^−/−^ mice [128]. *Cldn5* also is expressed in cerebral microvascular endothelial cells, perhaps regulating blood-brain barrier permeability [124, 130]. *Cldn5*^−/−^ mice develop normally but die shortly after birth [130]. There is no evidence for disrupted tight junction structure; however, small molecule paracellular permeability increases selectively in the mutant brain suggesting that lack of Cldn5 alters junctional function [130]. *CLDN5* is reduced in human end-stage cardiomyopathy [131, 132]. In addition, *CLDN5* is implicated in stem cell self-renewal. *ZO*-*1*, *CLDN1*, *CLDN3*, and *CLDN5* are highly expressed in human neural stem cells, but significantly down-regulated during neuronal differentiation [133].

*DGCR2 DiGeorge Syndrome Critical Region Gene 2* (a.k.a. *SEZ12*; **A to B**) encodes a novel putative transmembrane adhesion receptor [134] expressed in the developing and adult mouse brain (Fig. 1c) [58]. Recent studies support a role for *Dgcr2* in cortical projection neuron migration and differentiation, apparently as part of a complex that regulates Reelin-dependent phosphorylation of Dab1, Akt, Erk1/2 and subsequent signaling targets [135]. *Dgcr2* deficient mice have gait abnormalities, reduced movement, and impaired motor coordination possibly due to loss of cerebellar Purkinje cells [136]. Finally, a *DGCR2* missense mutation has been associated with non-syndromic SCZ [137], reinforcing a potential contribution to neural development or function.

#### Perspective: cell-cell adhesion genes

The **A to B** cell-cell adhesion genes, *ARVCF* and *CLDN5*, belong to key junctional complex gene families: catenin/cadherins and claudin/ZO-1, respectively. Based upon their **A to B** location and essential roles in adhesion, cell-cell interactions, morphogenesis, and maintenance of tissue integrity—all compromised in 22q11DS—they are likely, but as yet minimally investigated suspects for core phenotypes. Their fundamental functions assessed in vitro or by genetic manipulation in frog, zebrafish, and mouse are consistent with contributions to cardiac and neural anomalies. *DGCR2*, a proposed **A to B** SCZ vulnerability gene, is an apparent adhesion receptor involved in cortical neuronal migration. Diminished dosage of **A to B** adhesion genes may converge on broader signaling networks at 22q11DS phenotypic sites, or each gene may independently compromise development, differentiation, or adult function. Given the morphogenetic consequences of disrupted adhesion, the contributions of **A to B** cell adhesion gene dosage in 22q11DS pathogenesis merit additional genetic and functional analysis.

#### Protein trafficking

*SEPT5 Septin 5* (a.k.a. *CDCREL1* or *PNUTL1*; **A to B**) is a member of the highly conserved Septin family of GTP-binding proteins that act as dynamic, multifunctional protein scaffolds. First discovered as essential for cytokinesis in yeast, Septins are found in most eukaryotes except plants [138, 139]. They are found in protein complexes that regulate membrane dynamics, vesicle trafficking, cell cycle, cytokinesis, cytoskeletal reorganization, cell polarity, oncogenesis, and apoptosis [140]. SEPT5, expressed predominantly in neurons, was first isolated as a constituent of a synaptophysin-associated synaptic vesicle protein complex (Fig. 1c, d) [141, 142]. It localizes to presynaptic terminals, interacts with the SNARE protein Syntaxin, and negatively regulates exocytosis and neurotransmitter release [141, 143, 144]. Moreover, in mouse, Sept5 in the auditory calices of Held regulates the coupling of Ca^++^ influx to presynaptic neurotransmitter release, and its overexpression inhibits dopamine release in transfected neurons in vitro [145, 146]. Nevertheless, synaptic transmission is not disrupted in *Sept5*^−/−^ or *Sept3*^−/−^/*Sept5*^−/−^ mice, suggesting compensation by other family members [147, 148]. In mouse and rat, altered *Sept5* levels are linked to defective social interactions and cognitive impairment [149–152]. In addition, homozygous deletion of *SEPT5* and the adjacent *GP1BB* has been associated with cortical dysplasia with polymicrogyria, developmental delay, platelet secretion defects, social/emotional, and language/speech deficits [153].

*CLTCL1 Clathrin Heavy Chain Like 1* (a.k.a. *CHC22*; **A to B**) is a member of the *CLATHRIN* gene family crucial for intracellular endosome trafficking [154, 155]. Its potential function has been difficult to assess—it does not have a murine orthologue [156]. *CLTCL1* is expressed in all cell types; however, levels differ between tissues and developmental stages [157, 158]. It is expressed maximally during human prenatal brain development and declines significantly by early childhood—correlated with critical periods for neural circuit differentiation [159]. *CLTCL1* homozygous missense mutations are associated with a recessive disorder characterized by touch and pain insensitivity and severe intellectual disability [159]. In a human neural crest-derived cell line—relevant because nociceptive (pain) neurons are derived from the neural crest [160]—*CLTCL1* downregulation induces neuronal differentiation and facilitates RA-mediated neurite outgrowth [159]. *CLTCL1* is highly expressed in skeletal muscle where it modulates glucose transporter 4 intracellular trafficking [157], myogenesis, and repair [161, 162]. Neuronal and myogenic activities implicate *CLTCL1* as a candidate for 22q11DS dosage-dependent phenotypes. The lack of an animal model to test hypotheses of organismal function complicates further assessment of *CLTCL1*’s potential contributions to the 22q11DS phenotypic spectrum.

**Fig. 2:**
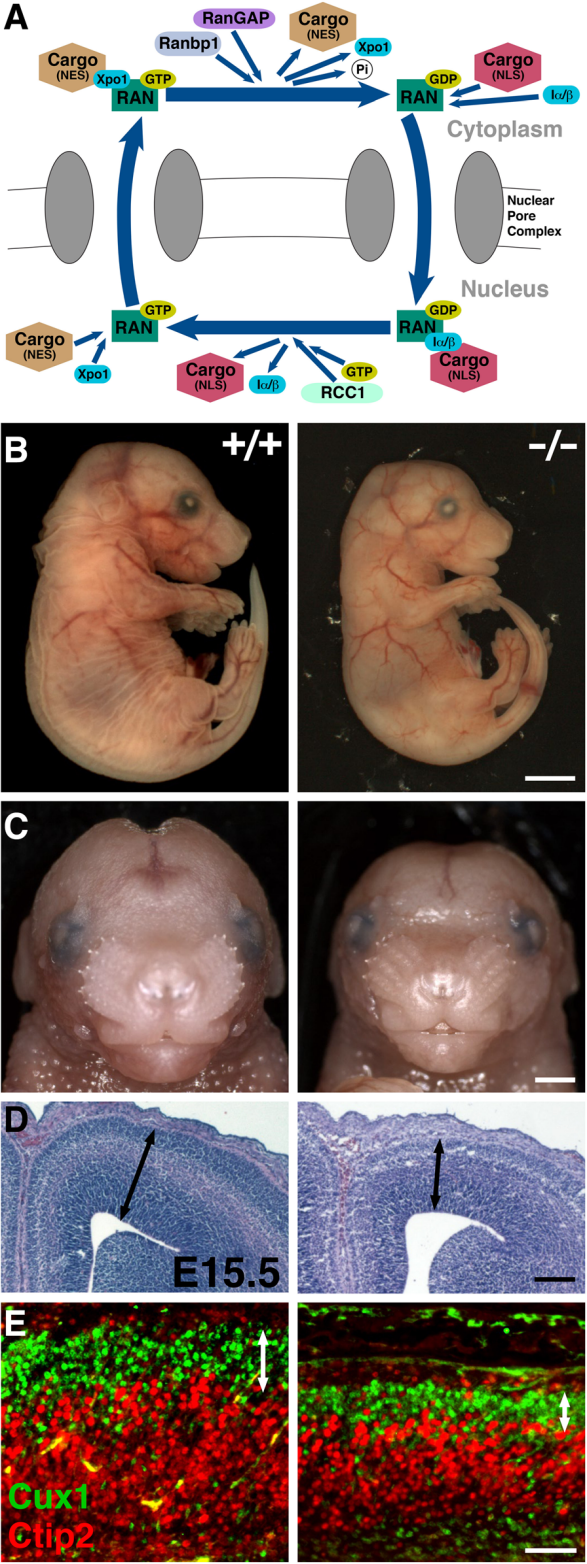
Ranbp1: function, craniofacial and neural phenotypes. **a** In the nucleus, the exportin (Xpo1) binds the cargo tagged with nuclear export signals (NES) and Ran-GTP and diffuses through the pore to the cytoplasm. Once in the cytoplasm, Ranbp1 along with RanGAPs that hydrolyze Ran-GTP into Ran-GDP dissociate the complex and release the transported macromolecule. In the cytoplasm, importin-α/β heterodimer forms a complex with cytoplasmic cargos tagged with a nuclear localization sequence (NLS) and Ran-GDP and transports them into the nucleus. Inside the nucleus, the high concentration of Ran-GTP rapidly binds to importin-β to dissociate the complex, releasing the transported cargos, followed by recycling of the importins back to the cytoplasm. RCC (a guanine nucleotide exchange factor) stimulates Ran-GDP to release its GDP and pick up GTP [164]. **b**, **c** Examination of E17.5 embryos shows that *Ranbp1*^−/−^ embryos have visible abnormalities including a smaller and shorter head with a narrower face*.* **d** Coronal sections show a distinctive smaller, thinner cortex in E15.5 *Ranbp1*^−/−^ embryos. **e** Layer 2/3 projection neurons, labeled for Cux1, are disrupted in E17.5 *Ranbp1*^−/−^ cortex [170]. Cux1 and Ctip2 labels subsets of upper- and lower-layer projection neurons, respectively. Scale bars: **b** = 250 μm, **c** = 100 μm, **d** = 25 μm, **e** = 50 μm

*RANBP1 RAN Binding Protein 1* (**A to B**) regulates the multi-protein “RAN complex” [163, 164]. The Ran complex includes the small G protein Ran, which when bound to GDP scaffolds other proteins including importins (Iα/β) that bind nuclear transport cargos (Fig. 2a). In the nucleus, the guanine exchange factor RCC1 replaces GDP bound to RAN with GTP, establishing new exportin (Xpo1) complexes that scaffold outgoing cargo. RANBP1 binds to the RAN complex as cargos exit the nucleus, displacing and releasing them into the cytoplasm and facilitating RanGAP GTPase activity [165]. RANBP1 also modulates mitotic spindle formation [166], nuclear envelope disassembly/reassembly [167], and primary cilia protein trafficking [168]. *Ranbp1* is expressed in neural crest and neocortical ventricular precursors at mid-gestation (Fig. 1c, d) [169, 170]. *Ranbp1*^−/−^ mice are not viable [170]. Over 50% of these embryos are exencephalic. Non-exencephalic homozygotes are smaller than WT littermates (Fig. 2b) with craniofacial and ocular anomalies (Fig. 2c). *Ranbp1* remains highly expressed in cerebral cortical ventricular and subventricular proliferative zones [58, 60, 170]. Non-exencephalic *Ranbp1*^−/−^ fetuses are microcephalic (Fig. 2c) with diminished cortical thickness (Fig. 2d, e). *RANBP1* haploinsufficiency has not been linked to single 22q11DS phenotypes; polymorphisms are associated with SCZ-related eye tracking anomalies [171] and reduced cortical expression is linked to disrupted metabotropic glutamate receptor signaling in ASD [172].

*SNAP29 Synaptosome Associated Protein 29 kDa* (**B to D**) belongs to the SNAP25 gene family encoding T-SNAREs that mediate vesicle/target membrane fusion [173]. SNAP29 co-localizes with endosomes, lysosomes, and Golgi in non-neuronal cells and influences protein trafficking, endocytosis, exocytosis, and phagocytosis [174–178]. Synaptic SNAP29 modulates neurotransmitter release by inhibiting SNARE complex disassembly [179, 180]. Snap29 also interacts with GTPase Rab3A in myelinating glia and is implicated in surface-directed myelin proteolipid protein trafficking [181]. *SNAP29* loss-of-function, independent of 22q11DS, is linked to CEDNIK: Cerebral Dysgenesis, Neuropathy, Ichthyosis, and Keratoderma [182, 183], characterized by psychomotor retardation, thick, scaly skin, facial anomalies, sensorineural deafness, microcephaly, corpus callosum dysgenesis, and cortical dysplasia. Parallel brain and epidermal phenotypes in constitutive and conditional *Snap2*^−/−^ mice [184] reinforces its potentially causal role in CEDNIK. Its contributions to 22q11DS phenotypes in typically deleted individuals (**A to D**), however, remain unanalyzed.

#### Perspective: protein trafficking genes

Disrupted protein trafficking due to diminished 22q11.2 gene dosage could have significant effects on signaling during development, differentiation, or in the mature nervous system and other 22q11DS phenotypic sites. *CLCTL1*, *RANBP1* (**A to B**), and *SNAP29* (**B to D**) are required for early brain development; *SEPT5* (**A to B**) is not. Instead, in mice, *Sept5* may modulate circuit development or neuronal signaling for cognitive and social behaviors. Evidence for *Ranbp1*-dependent regulation of neural and craniofacial differentiation is fairly strong. It is unclear, however, how diminished *RANBP1* dosage interacts with other **A to B** or **B to D** genes to yield 22q11DS phenotypes. *SNAP29* may modulate **A to B** dependent craniofacial development due to its activity in neural crest, or dosage sensitivity in non-neural crest cells that contribute to facial structures. Accordingly, based upon obligate and disease-related functions, *SNAP29* may act as a modifier for some 22q11DS phenotypes.

#### mRNA/miRNA biogenesis and processing genes

*DGCR14 DiGeorge Critical Region Gene 14* (a.k.a. *ESS2* or *DGSI*; **A to B**) encodes a nuclear protein with two N-terminal coiled-coil domains [185] and is conserved across eukaryotes from yeast to human [186–189]. In mice, *Dgcr14* is expressed in the pharyngeal arches and neural tube [186]. Despite its expression at key sites, the consequences of loss-of-function mutations in this gene have not been reported in mice or any other model species. *Dgcr14* expression has been assessed in the context of loss of function of another **A to B** gene, *Gsc2* (see below), whose expression is limited to the interpeduncular nucleus (IPN)—a brain region that modulates dopaminergic neurotransmission, sleep, and eye movement. *Dgcr14* expression, also enhanced in the IPN, is diminished following *Gsc2* deletion [190]. The significance of this observation for *Dgcr14* function or that of *Gsc2* in the IPN, however, remains unexplored. A recent large-scale protein interactome study identified DGCR14 as a non-core component of the spliceosome C complex, suggesting a role in mRNA processing [191–193]. Indeed, *Dgcr14* apparently regulates *IL17a* transcription during TH17 cell differentiation in vitro [194], perhaps relevant for immune dysfunction in 22q11DS. In human, *DGCR14* is expressed in the heart, brain, and skeletal muscle [195]. The exact function of *DGCR14* in human development or mature function, however, remains uncertain.

*DGCR8 DiGeorge Critical Region Gene 8* (a.k.a. *Pasha*; **A to B**) encodes a heme-binding protein known as Pasha in flies and nematodes [196]. DGCR8 is localized to the nucleus as part of the Drosha/RNaseIII microprocessor complex that processes primary microRNAs (pri-miRNA) into precursor miRNA (pre-miRNA) in the first step of miRNA maturation [196, 197]. DGCR8 also has a microprocessor-independent role in small nucleolar RNAs (snoRNAs) and telomerase RNAs biogenesis [198]. DGCR8 is required for normal development of the nervous system [199, 200]. In *Drosophila*, *pasha* influences dendrite targeting and axon terminal arborization [201, 202]. In mice, *Dgcr8* is highly expressed in the brain (Fig. 1c) and loss-of-function results in pre-implantation lethality [200]. *Dgcr8* heterozygous deletion, however, results in working memory deficits and impaired short-term synaptic plasticity due to defective dendritic spines and complexity [200, 203]. Furthermore, *Dgcr8* targeted deletion in pyramidal neurons alters synaptic transmission [204, 205]. *Dgcr8* deficiency also has been suggested to underlie aberrant cortical interneuron migration caused by disrupted Cxcr4/Cxcl12 signaling [206, 207]. *DGCR8* may modulate gene expression underlying establishment and maintenance of peripheral myelination [208]. Targeted *Dgcr8* loss-of-function in cardiac neural crest leads to persistent truncus arteriosus, ventricular septal defects [209], cardiomyocyte-related cardiomyopathy, and premature lethality [210].

#### Perspective: mRNA/miRNA biogenesis and processing genes

The **A to B** location of miRNA biogenesis 22q11.2 genes *DGCR14* and *DGCR8*, acknowledged functional importance of this miRNA regulation of development and homeostasis, and requirement for *DGCR14* and *DGCR8* for nervous system and heart development establishes these two genes as prime suspects for understanding 22q11DS phenotypes and their variability. A circumstantial case can be made for *DGCR14* function in the nervous and immune systems; however, there is still surprisingly little known about indispensable functions of this gene. The contributions of *DGCR8* to 22q11DS are beyond doubt. Nevertheless, *Dgcr8* heterozygous phenotypes and those of mice with full deletion of the **A to B** orthologue are not identical [26]. Thus, key DGCR8 functions remain to be explored further in the context of diminished dosage of additional 22q11.2 **A to B** as well as **B to D** genes.

#### Transcription factors

**Fig. 3:**
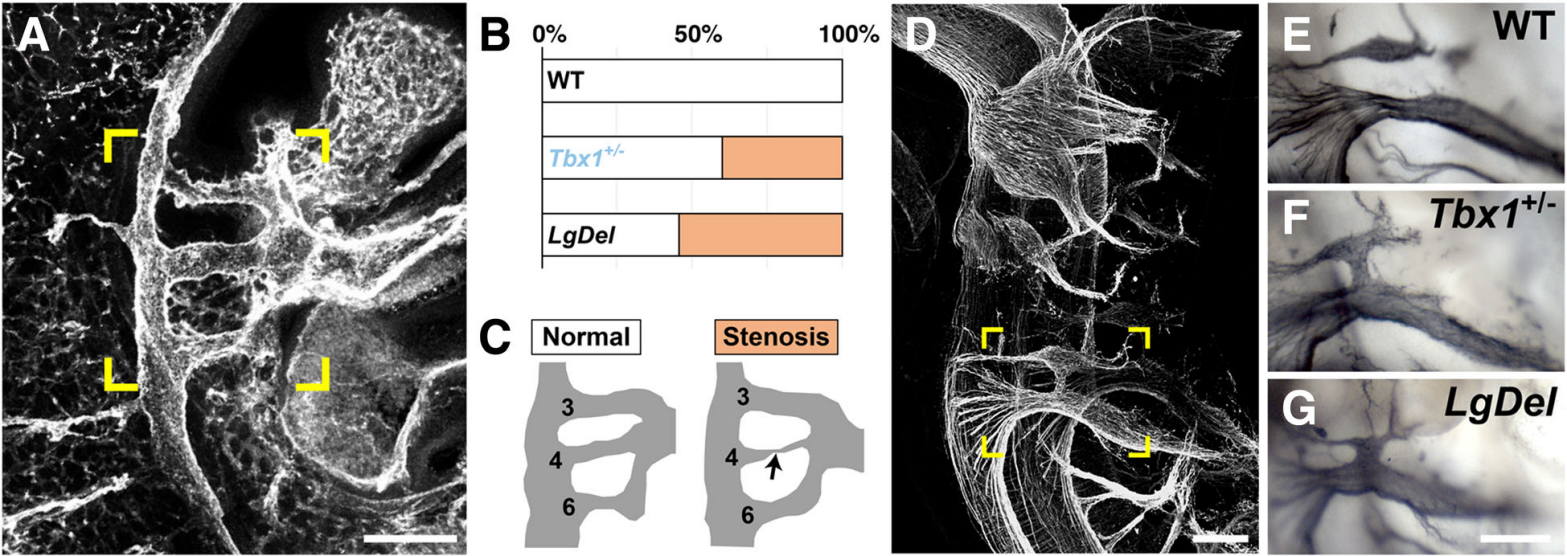
Tbx1: heart and cranial nerve phenotypes. **a** The heart and pharyngeal arch arteries in an E10.5 WT embryo, stained for the cell-adhesion molecule PECAM/CD31 and imaged whole. **b** The graph indicates the % stenosis in WT, *Tbx1*^+/−^, and *LgDel* embryos [73]. **c** A schematic the arch vasculature is provided for clarity. **d** Lateral view of neurofilament labeled E10.5 cranial nerves in a *Tbx1*^+/−^ embryo (CNs). **e**, **f** The ganglia of CNIX and CNX are more frequently fused or connected by axon fascicles in *Tbx1*^+/−^ than WT littermates. **g** CNIX/X fusion is observed at similar frequency in *LgDel* embryos [74]. Scale bars: 200 μm

*TBX1* (**A to B**) is a member of the “T-box” family of transcription factors. Although *TBX1* is neither a strong transcriptional activator nor a strong repressor, it regulates a large number of genes through epigenetic modifications [211]. *Tbx1* is expressed in pharyngeal arch endoderm and mesoderm, an expression pattern conserved in mice, chickens, *Xenopus*, and zebrafish [212–214]. A great deal of insight—and speculation—has arisen from analyses of *Tbx1* loss-and gain-of-function mutants in a variety of model species. In zebrafish, the *tbx1* loss-of-function *van gogh* mutation results in ear, thymus, and pharyngeal arch defects [214]. In *Xenopus*, its loss-of-function disrupts head, pharyngeal apparatus, and heart development [212, 215]. *Tbx1* heterozygous mutant mice have mild non-lethal phenotypes; homozygous null mutants, however, die at birth with cardiac outflow tract anomalies, craniofacial defects, cleft palate, and severe thymus and parathyroid abnormalities [216–218]. In mouse models, the narrowing (stenosis) of the fourth pharyngeal arch artery that develops into the central portion of the aortic arch is the most prominent cardiovascular phenotype (Fig. 3a–c) [74]. Complete *Tbx1* inactivation in pharyngeal endoderm is sufficient to cause a phenotype identical to the *Tbx1* null mutants [219]. Part of the cardiovascular defect in *Tbx1*^−/−^ mice reflects failed cardiac neural crest migration into the pharyngeal arches and heart where the crest produces signals such as RA that pattern heart structures [220]. *Tbx1* in the otic vesicle is required for inner ear development, and also is essential for face and limb myoblast differentiation [213, 221–223]. FGF signaling apparently influences *Tbx1* function for pharyngeal arch derivatives [224]. *Tbx1* heterozygosity is partially responsible for cranial nerve dysmorphology in the *LgDel* model of 22q11DS (Fig. 3d–g) [74]. *Tbx1* heterozygotes have a significantly higher frequency of fusions and anastomoses between the glossopharyngeal (IX) and vagus (X) cranial nerves than WT embryos (Fig. 3e, f) [74]. This frequency is equivalent to that in *LgDel* embryos (Fig. 3d); however, *Tbx1* mutants do not display the RA-sensitive *LgDel* trigeminal nerve (V) phenotype [74]. *TBX1* heterozygous mono-allelic mutations have been associated with conotruncal anomaly face syndrome, thymic hypotrophy, parathyroid dysfunction, and deafness in a small sample of individuals *without* broader 22q11.2 deletion, providing a further suggestion of a key role in individuals *with* broader 22q11.2 deletion [225]. Finally, heterozygous *Tbx1* mutation, like *Gnb1l* (an **A to B** protein trafficking gene; see above), reduces PPI in mice, indicating possible involvement in behavioral disorders [64]. This phenotypic equivalence, however, raises additional questions regarding the specificity of the PPI assay as well as a singular role for *Tbx1* (or *Gnb1l*) in complex phenotypes, either in 22q11DS, or a wide range of animal models.

*GSC2 Goosecoid Homeobox 2* (a.k.a. *Goosecoid-like*, *GSCL*; **A to B**) encodes a homeodomain-containing protein [226]. It is related to the homeobox gene, *Goosecoid*, which is required for craniofacial and rib development in mice [227]. Mouse *Gsc2* is expressed in the neural tube and pharyngeal arches during neural crest migration and differentiation [228]. Nevertheless, homozygous *Gsc2* mutant mice are viable with no obvious developmental anomalies [229]. As mentioned above, *Gsc2* expression in the mature nervous system is restricted to the midbrain interpeduncular nucleus (IPN), implicated in dopaminergic modulation, sleep, and rapid eye movements. Despite this restricted expression, IPN-related phenotypes have not been reported in *Gsc2*^−/−^ mice, except for loss of expression of an additional 22q11.2 **A to B** gene, the miRNA biogenesis factor *Dgcr14* (see above) [190].

*MED15 Mediator Complex Subunit 15* (a.k.a. *ARC105*; **B to D**) encodes a subunit of the mediator complex, a cofactor for RNA Polymerase II-dependent transcription. MED15 acts within the pre-initiation complex, which consists of MED15, POLII, TFIIA, TFIIB, TFIID, TFIIE, TFIIF, and TFIIH, and is considered a global regulator of gene expression [230, 231]. Mediator complex genes encode up to 30 subunits in some eukaryotes [231], and have limited homology between species. The MED15 subunit was initially identified based upon homology with Gal11, an essential transcriptional regulator of galactose metabolism in yeast [232], in which it may also function as a regulator of lipid homeostasis [233]. The *C*. *elegans* MED15 homolog, *mdt-15* is required for fatty acid metabolism via transcription of genes that modulate desaturation of stearic to oleic acid [234]. *Mdt*-*15* deficient worms move abnormally, are sterile, are more sensitive to stress, have disrupted ER homeostasis, and shortened life spans [234–236]. In *Drosophila*, *med15* is required for the transcription of *decapentaplegic* (*dpp*, homolog of vertebrate bone morphogenetic proteins (BMPs)) target genes [237]. The *Xenopus* MED15 homolog, *arc105*, similarly regulates *transforming growth factor beta* (*TGFβ*)/*Activin*/*Nodal*/*Smad2*/*3* target gene expression [238]. MED15 function during mammalian development, however, remains elusive—there are no reported mouse mutants, nor are there any clinically described *MED15* mutations, independent of heterozygous 22q11.2 **A to D** deletion, in humans.

*KLHL22 Kelch Like Family Member 22* (**B to D**) encodes a member of the Bric-a-brac-Tramtrack-Broad complex (BTB)-*Kelch* transcription factor family that is conserved from *Drosophila* to humans [239, 240]. The BTB-Kelch proteins contain a BTB/POZ domain, a BACK domain, and five to seven Kelch motifs [239]. The BTB domains facilitate protein binding and have multiple cellular roles including recruitment of E3 ubiquitin ligase complex [241–243]. Kelch domains form a β-propeller structure that, at least in some cases, interacts with actin and intermediate filaments and is involved in cytoskeleton organization [239, 244]. The BACK domain is a conserved motif found in the majority of proteins that contain both BTB and Kelch domains. Although no function has been reported for the BACK domain, it is likely to be of functional significance because mutations in this motif have been linked to human axonal neuropathy [245].

*LZTR1 Leucine-Zipper-Like Transcription Regulator 1* (**B to D**) also encodes a member of the BTB-Kelch superfamily [244]. LZTR1 was initially described as a transcriptional regulator based on a weak homology to members of the basic leucine zipper-like family. However, further studies showed that LZTR1 localizes exclusively to the Golgi network where it helps stabilize the Golgi complex [246]. *LZTR1* mutations are related to a rare genetic disorder called Schwannomatosis, characterized by multiple intracranial, spinal, and peripheral tumors (schwannomas) [247, 248]. Furthermore, biallelic pathogenic variants in *LZTR1* have been linked to Noonan syndrome, a disorder characterized by unusual facial features, short stature, cardiovascular anomalies, bleeding, skeletal malformations, and developmental delays [249]. The parallels of Noonan/*LZTR1* loss of function phenotypes with those in 22q11DS suggest it is a viable candidate for modulating core 22q11.2 anomalies in individuals with 3 Mb **A to D** deletions.

*ZNF74 Zinc Finger Protein 74* (**B to D**) belongs to a large subfamily of C_2_-H_2_ (Cys_2_-His_2_) zinc finger proteins encoding Kruppel-associated box (KRAB) transcriptional repressor motifs [250]. In addition to DNA binding activity, a subset of the C_2_-H_2_ proteins bind RNAs and proteins [251, 252]. Family member TFIIIA binds to RNAs and proteins to regulate 5S rRNA transcription, storage, and transport from the nucleus to the cytoplasm [253, 254]. Similarly, *ZNF74* encodes a nuclear matrix-attached protein with RNA binding properties [255]. ZNF74 interacts with a hyper-phosphorylated form of the largest subunit of RNA polymerase II and may regulate its activity during pre-mRNA processing [256]. There are no mouse or human genetic or functional analyses of this gene***.***

*HIC2 Hypermethylated in Cancer 2* (a.k.a. *ZBTB30* and *ZNF907*; **B to D**) is a BTB-zinc finger transcription factor required for normal heart development [257]. Homozygous *Hic2* loss-of-function is early embryonic lethal and heterozygous mutants have ventricular septal defects and die at birth [258]. These anomalies implicate *HIC2* as a potential modulator of 22q11DS phenotypes, particularly those in the heart, in typically deleted (**A to D**) individuals.

*THAP7 Thanatos*-*Associated Protein 7* (**B to D**) encodes a member of a large family of THAP, an N-terminus 89-amino acid motif/C_2_-CH signature (Cys-X_2–4_-Cys-X_35–53_-Cys-X_2_-His) transcription factors [259]. THAP transcriptional regulators, found only in animal genomes [259, 260], are multifunctional transcriptional regulators. *C*. *elegans* THAP protein (HIM-17) is critical for chromosome segregation during meiosis [260]. The C_2_-CH signature zinc finger domain in *THAP7* is similar to the site-specific DNA binding domain of *Drosophila* P-element transposase [259]. Human THAP7 functions as a transcriptional repressor by recruiting co-repressor NcoR and histone deacetylase HDAC3 to chromatin [261, 262]. Despite these intriguing functions, little is known about potential *THAP7* contributions, based upon human mutations or animal models, to 22q11DS phenotypes.

#### Perspective: transcription factors

There are eight transcription factors located between LCRA and LCRD on hChr22q11.2: two in the **A to B** region, and six in the **B to D** region. Of these eight, *TBX1* (**A to B**), *HIC2* (**B to D**), and *LZTR1* (**B to D**) when mutated in mice or humans influence heart and craniofacial phenotypes that have similarities to those in 22q11DS. Of all transcription factors, *TBX1* has received the most attention as a “candidate” or even a single explanatory gene for 22q11DS phenotypes. *TBX1* makes an essential contribution to 22q11DS cardiac, craniofacial, and otic phenotypes. Whether these contributions distinguish it formally as haploinsufficient for subsets of 22q11DS heart and face phenotypes versus a modulator of additional **A to B** or **B to D** gene interactions remains unresolved [73, 74]. Evidence for *TBX1* modulation of brain development, particularly phenotypes crucial for 22q11DS behavioral deficits [211, 263] also is unresolved, although it is likely more limited than its contributions to cardiac or craniofacial differentiation [26]. In mouse, *Tbx1* regulates distinct aspects of morphogenesis of cranial nerves IX and X (glossopharyngeal and vagal) that participate in respiratory control, feeding, and swallowing, suggesting that *TBX1* may contribute to abnormalities in swallowing behavior observed in many individuals with 22q11DS [74, 264]. *GSC2* (**A to B**) is not a likely contributor to 22q11DS pathology based upon available evidence. The role for dosage-dependent modulation of “core” (**A to B**-related) 22q11DS phenotypes of the remaining five **B to D** transcription factors could be significant, based on their apparent far-reaching effects on development and function. Nevertheless, almost no data, as yet, address possible interactions between modifying **B to D** transcription factors critical **A to B** genes.

#### Transmembrane receptors/transporters

*RTN4R Reticulon 4 Receptor* (a.k.a. *Nogo* receptor, *Nogo-66* receptor, or *NGR1*; **A to B**). Of all the **A to B** genes, *RTN4R* is an intriguing candidate for nervous system phenotypes, and perhaps the most controversial. *RTN4R*, a neuronal glycosylphosphatidylinositol (GPI)-linked receptor, is expressed mainly in cerebral cortical neurons, hippocampal neurons, cerebellar Purkinje cells, and pontine neurons [265]. RTN4R binds to myelin-derived inhibitory proteins, MAG, OMgp, and Nogo isoforms and may limit neurite growth and axon regeneration after CNS injury in adult mammals [266–269]. Consistent with these observations, in vivo studies report improved axon growth and locomotor recovery in spinal cord injured *Rtn4r*^−/−^ mice or in injured rats treated with anti-Nogo-A antibodies [268, 270]. Adult rat motor cortical neurons can functionally reorganize after injury and treatment with anti-Nogo antibody, accompanied by increased movement of the lesion-impaired forelimb [271]. In the hippocampus, Rtnr4 restricts formation of both excitatory and inhibitory synapses, influences dendrite spine morphology and limits activity-dependent synaptic strength [272–275]. In the visual cortex, Rtnr4 consolidates neural circuitry established during the visual critical period, and in other forebrain regions, it is required for consolidation of long-term memory [276–278]. These results, however, have been challenged. Indeed, they have not been fully replicated and a great deal of evidence suggests that the Nogo/Rtn4r pathway alone makes at best a modest contribution to mammalian central nervous system regeneration and repair [279–281]. In addition to its adult CNS expression, *Rtn4r* is expressed during brain development [282]. Nogo-A signaling via Rtn4r is implicated in ES cell self-renewal and differentiation [283–285]. It may influence neural precursor migration in the developing and adult cerebral cortex [282, 286]. *RTN4R* also has been implicated as a susceptibility gene in psychosis through disruption of the brain microstructure [287, 288]. Several recent in vitro studies detect *Rtn4r* in immune cells, suggesting regulatory functions in immune cell adhesion and migration [289–291]. The lack of clear data defining the function of Nogo-A/Rtn4r signaling makes its contribution to 22q11DS phenotypes uncertain.

*P2RX6 Purinergic 2X6 Receptor* (**B to D**) encodes a member of the P2X purinergic receptor family, which are ligand-gated ion channels activated by extracellular ATP, a neurotransmitter that causes fast excitatory postsynaptic potentials in neurons and smooth muscles [292–295]. P2X receptors are trimers of P2X subunit proteins (P2X1–7) that associate as homomers or heteromers. The P2X6 subunit only assembles with P2X2 or P2X4 to form an active receptor complex [296, 297]. The *P2rx6* gene was first isolated from a cDNA library from rat superior cervical ganglion cells [298], which are derived from the neural crest [299]. *P2rx6* is expressed in the brain, localizes to glutamatergic synapses, increases during post-natal neurogenesis [300–302], and modulates neuronal differentiation from neural progenitors in vitro [303]. Despite cellular evidence for *P2rx6* function throughout the murine nervous system, there is no evidence for the effect of *P2RX6* deficiency or null mutation either on mouse or human brain development.

*SLC7A4 Solute Carrier Family 7 Member 4* (*a.k.a. CAT4*; **B to D**) is related by sequence to the cationic amino acid transporters (CAT) family [304]. In spite of its localization in the plasma membrane, *SLC7A4* does not have transport activity when overexpressed in human cells or in *Xenopus* oocytes [305].

#### Perspective: transmembrane receptors/transporters

The one transmembrane receptor in the **A to B** region, *RTN4R* or *NogoR*, has potential nervous system functions that may contribute to 22q11DS phenotypes. Nevertheless, mouse heterozygous or homozygous mutants do not have reported phenotypes that parallel any of those associated with individuals with 22q11DS or mice with the orthologous **A to B** deletion [268, 280, 306]. Despite the still uncertain function of *RTNR4* in neuronal plasticity or regeneration, it may interact with other **A to B** or **B to D** genes to modify 22q11DS phenotypes, particularly in the nervous system. Of the two **B to D** receptors, the *P2RX6* is the most viable candidate for additional 22q11DS phenotypic variability.

#### Mitochondrial genes

**Fig. 4:**
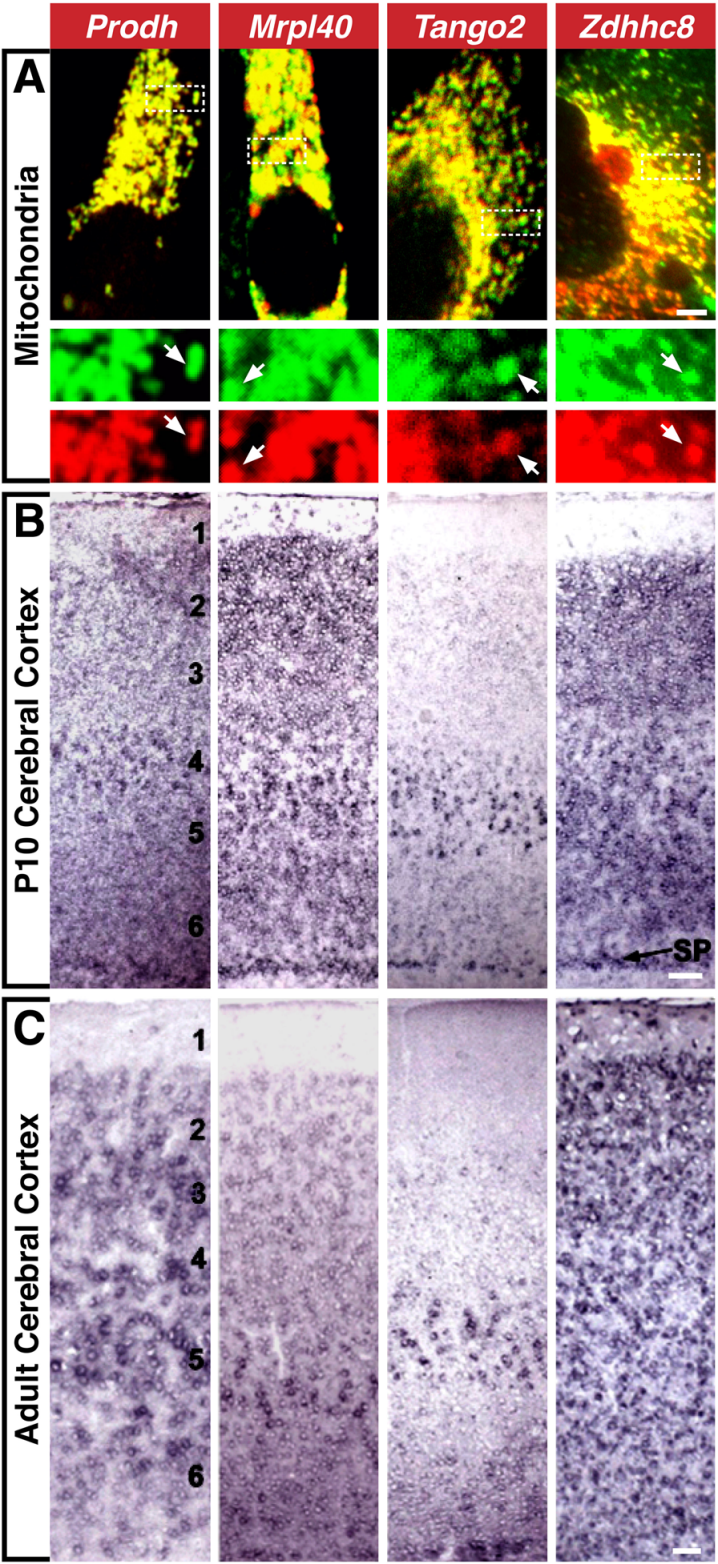
Mitochondrial genes. **a** Co-localization of Prodh, Mrpl40, Tango, and Zdhhc8-GFP fusion proteins (green) and a mitochondria-targeted mCherry reporter (red). Inset: separated red and green channels. **b** and **c** *Prodh*, *Mrpl40*, *Tango2*, and *Zdhhc8* expression patterns in P10 **b** and adult **c** cerebral cortex. 1–6 represent the six neocortical layers: 1, closest to the pia; 6, closest to the subcortical white matter. *SP* subplate [310]. Scale bars: **a** = 5 μm, inset = 2.66x, **b**, **c** = 50 μm

*PRODH Proline Dehydrogenase* (a.k.a. *Proline Oxidase*; **A to B**) encodes a mitochondrial gene that catalyzes the first step of proline to glutamate conversion (Fig. 4a) [307, 308]. In the brain, *Prodh* is selectively expressed in the hypothalamus, amygdala, piriform cortex, hippocampus, and cerebral cortex (Figs. 1c and 4b, c) [58, 309–311]. PRODH may be key for synaptic transmission. Proline may act as a neurotransmitter; however, the lack of a specific receptor in the brain suggests it may function instead as a neuromodulator [312, 313]. Indeed, in the mouse brain, proline may modulate glutamatergic synaptic transmission by directly activating glutamate receptors or potentiating transmission [314–318]. In hyperprolinemic mice, memory deficits accompany elevated proline levels [319–321]. *Prodh*^−/−^ mice have sensorimotor gating defects and decreased hypothalamic glutamate and GABA [322]. *PRODH* loss of function mutations results in hyperprolinemia type 1, whose clinical symptoms largely depend on how severely proline function is reduced [307, 323]; while a minor increase in proline is mostly considered benign, high accumulation of proline is associated with severe phenotypes [323]. SCZ, intellectual disability, severe speech disorders, and seizures are associated with reduced PRODH activity due to polymorphisms or biallelic mutations independent of 22q11.2 deletion [323–326]. A genome-wide association study, however, has challenged PRODH’s role as a risk factor for SCZ [327].

*MRPL40 Mitochondrial Ribosomal Protein L40* (a.k.a. *NLVCF*; **A to B**) localizes to mitochondria and encodes the large subunit of the mitochondrial ribosome (Fig. 4a) [328]. *Mrpl40* is expressed in the first and second pharyngeal arches in the mouse [329]. In the post-natal brain, *Mrpl40* is enhanced in olfactory bulb mitral cells, cerebellar Purkinje cells, layer 5/6 cerebral cortex projection neurons, and the cortical subplate (Fig. 4b) [310], a transient early generated neuronal population between layer 5/6 and the cerebral ventricle that is necessary for initial thalamo-cortical and cortico-thalamic circuit formation [330, 331]. In the adult, *Mrpl40* is enhanced in the same projection neuron classes (Fig. 4c) [310]. Heterozygous *Mrpl40* mutants have synaptic plasticity and working memory deficits, altered mitochondrial permeability, increased presynaptic cytosolic Ca^+^^+^, and enhanced short-term potentiation [332].

*TANGO2 Transport and Golgi Organization 2 Homolog* (a.k.a. *T10*; **A to B**) encodes a member of the transport and Golgi organization family that plays roles in ER secretory protein loading [333]. In *Drosophila*, *tango2* loss-of-function leads to Golgi/ER fusion [333]. In contrast, mouse *Tango2* has a mitochondrial localization motif and localizes to mitochondria, but not Golgi or peroxisomes (Fig. 4a) [310]. In the early postnatal mouse brain, *Tango2* is restricted to olfactory bulb mitral cells (OB projection neurons), layer 5/6 cortical projection neurons, and the cortical subplate [310], and this restricted expression is maintained in the adult, except for the transient subplate (Fig. 4c). Mouse *Tango2* mutants have not been reported. However, *TANGO2* biallelic truncation mutations have been linked to a metabolic disorder, infancy-onset episodic metabolic crisis [334, 335], characterized by muscle weakness, recurrent rhabdomyolysis, cardiac arrhythmia, encephalopathy, progressive neurodegeneration, and cognitive impairment. In fibroblasts from an individual with a *TANGO2* mutation, ER stress increases, Golgi volume and density decline, and mitochondrial fatty acid β-oxidation is aberrant [335]. Apparently, TANGO2 merits further assessment in humans and model systems as a contributor to the 22q11DS phenotypic spectrum.

*ZDHHC8 Zinc Finger DHHC-Type Containing 8* (**A to B**) encodes a putative member of a family of transmembrane palmitoyl-transferases that share a conserved cysteine-rich catalytic domain (DHHC) [336]. It may modify multiple substrates including cell adhesion molecules, ion channel components, signaling, scaffold, and membrane-associated proteins [336]. Zdhhc8 has a mitochondrial signal sequence and localizes to mitochondria (Fig. 4a) [310]. In developing and adult mouse brain, *Zdhhhc8* is enriched in the olfactory bulb, hippocampus, cerebellum, thalamic relay nuclei, and cerebral cortex (Figs. 1c, d and 4b, c) [58, 310, 311], and the protein is restricted to synapses in all of these regions. *Zdhhc8* null mice are viable; however, hippocampal and cortical dendrite and axon growth as well as arborization, spine density, and glutamatergic synapse frequency declines [337, 338]. Some of these deficits can be rescued by enzymatically active ZDHHC8 [338]. Human ZDHHC8 palmitoylates PSD95, an adaptor involved in excitatory synapse development and plasticity [337, 339–342] and PICK1, whose palmitoylation is necessary for cerebellar long-term synaptic depression [343]. ZDHHC8-dependent palmitoylation also is required for trafficking and stability of the D2 dopamine receptor protein to the plasma membrane [344]. ZDHHC8 is suggested to increase the risk of SCZ [345], although this claim was challenged in a recent study [327].

*SLC25A1 Solute Carrier Family 25 Member 1* (a.k.a. *CTP*; **A to B**) encodes a highly conserved inner mitochondrial membrane transporter that mediates the movement of citrate/isocitrate in exchange for cytosolic malate [346, 347]. It modulates mitochondrial homeostasis, fatty acid and sterol biosynthesis, glycolysis, chromosome maintenance, and cytokine-dependent inflammatory responses [346, 348–350]. In zebrafish, *slc25a1* knockdown results in neuromuscular junction anomalies, axon outgrowth defects, and synaptic abnormality [351]. In humans, biallelic *SLC25A1* mutations are associated with combined D-2- and L-2-hydroxyglutaric aciduria, a rare neurometabolic disorder characterized by hypotonia, epileptic encephalopathy, respiratory insufficiency, developmental arrest, severe neurodevelopmental dysfunction, lack of psychomotor development, and early death [352, 353]. Corpus callosum agenesis, optic nerve hypoplasia, and facial dysmorphism accompany these core clinical signs [354, 355]. *SLC25A1* hemizygous mutation in combination with 22q11.2 deletion has been reported in an individual with severe brain, heart, and face phenotypes along with combined D-2- and L-2-hydroxyglutaric aciduria [356].

*TXNRD2 Thioredoxin Reductase 2* (a.k.a. *TR3*; **A to B**) encodes a pyridine nucleotide-disulfide oxidoreductase family seleno-cysteine enzyme [357]. Txnrd2 mainly localizes to mitochondria; however, various N-terminal splicing variants may direct it to other cellular compartments [358]. Txnrd2 has two redox-active catalytic domains responsible for mitochondrial scavenging of reactive oxygen species essential for cell survival and mitochondria-mediated apoptosis [359–361]. During mouse development, *Txnrd2* is expressed in the heart and its constitutive loss-of-function leads to anemia, heart ventricular thinning, and fetal death [362]. Heart-specific *Txnrd2* inactivation also elicits heart chamber dilation, swollen, structurally altered mitochondria, and death shortly after birth [362], reminiscent of human dilated developmental cardiomyopathy [363]. Heart-specific *Txnrd2* inactivation in adult mice increases vulnerability to myocardial ischemia/reperfusion injury due to mitochondrial degeneration and contractile dysfunction [364, 365]. Even though oxidative stress is linked with neurodegeneration, nervous system-specific ablation of *Txnrd2* does not cause apparent gross abnormalities in any region of the mouse brain [366]. In contrast to other studies that support the critical role of selenium and selenoproteins in immune responses [367, 368], T cell- and B cell-specific *Txnrd2* disruption failed to phenocopy key defects in fetal hematopoiesis [369]. *Txnrd2* loss-of-function phenotypes are similar to clinical features of Keshan Disease, caused by severe selenium deficiency [370].

#### Perspective: mitochondrial genes

22q11.2 mitochondrial genes are found only in the **A to B** region. These six genes: *PRODH*, *MRPL40*, *TANGO2*, *ZDHHC8*, *SLC25A1*, and *TXNRD2* are implicated in cardiac, facial, and neural development, as well as brain function, and cognitive skills. The absence of **B to D** mitochondrial genes suggests 22q11DS bioenergetic/metabolic dysfunction may reflect primarily dosage change of the six **A to B** mitochondrial genes. Other **B to D** genes might modulate the functions of the six **A to B** mitochondrial genes, albeit indirectly. Nevertheless, single gene phenotypes in animal models and rare human homozygous loss-of-function mutations are all closely related to core 22q11DS phenotypes. Thus, a circumstantial case can be made for **A to B** mitochondrial gene contributions; however, this case needs to be strengthened by additional experimental and genetic evidence.

#### Hemostatic factors

*GP1BB Glycoprotein Ib Platelet Beta Subunit* (a.k.a. *CD42C*; **A to B**) encodes a subunit of the GP1b-V-IX complex, a hemostatic and thrombotic protein receptor primarily on platelet surfaces [371]. *GP1BB* mutations are associated with Bernard-Soulier syndrome [372], a rare disorder characterized by large platelets, low platelet count, and prolonged bleeding time [371]. Accordingly, there have been no assessments of *Gp1bb* mutant phenotypes in animal models.

*SERPIND1 Serpin Family D Member 1* (a.k.a. *Heparin Cofactor II*; **B to D**), a serpin inflammation and coagulation superfamily modulator [373, 374], is a serine protease inhibitor that rapidly inactivates thrombin, a coagulation-related protease, and promotes angiogenesis and vascular remodeling after injury [375, 376]. Polymorphisms on the *SERPIND1* promoter region may influence its expression [377]; however, no additional analyses confirm this finding.

#### Perspective: hemostatic factors

Hemostatic disruption has not been proposed as a frequent 22q11DS phenotype. Nevertheless, these anomalies are more common than previously thought [378–380]. The disruption is mostly linked to *GP1BB*; however, there is a recent report that challenges this correlation [381]. It also possible that *SERPIND1* located in **B to D** region modifies the function of *GP1BB* the in **A to D** deleted individuals, especially if regulatory polymorphisms further vary expression levels of this **B to D** gene.

#### Additional genes with unique functions

*DGCR 6 DiGeorge Critical Region 6* (**A to B**) is encoded by two highly homologous ORFs (*DGCR6* and *DGCR6L*) [382]. *DGCR6* has homology to *Gdl*, a gonadal development related protein in *Drosophila*, and the human laminin gamma-1 subunit, LAMC1, which is essential for basal lamina assembly [383]. Although distinct functions for the two ORFs encoding *DGCR6* variants have not yet been fully defined, *DGCR6* may influence neural crest migration and pharyngeal arch development [58, 384, 385]. In chick embryos, *DGCR6* suppression results in cardiovascular anomalies [384]. In these embryos, attenuation of *DGCR6* stimulates *TBX1* and *UFD1L* but decreases heart and pharyngeal arch *HIRA* expression (all **A to B** genes), suggesting it is a modifier of critical region phenotypes [384]. *DGCR6* also has been implicated in 22q11DS conotruncal heart defects, either directly or through *TBX1* activity [386]. Dysregulation of *DGCR6* and *DGCR6L* is suggested to be associated with neuropsychological findings in 22q11DS children [387].

*UFD1L Ubiquitin Fusion-Degradation Protein-1 Like* (a.k.a. *UFD1*; **A to B**) is a component of the Ufd1L-Npl4-p97 multiprotein complex that recognizes and presents several polyubiquitin-tagged proteins to the proteasome for degradation [388, 389]. IP3 receptors, downstream components of PI4KA pathway (see above), are included among Ufd1L-Npl4-p97 targets [390]. The Ufd1-Npl4-p97 complex is required for clearance of damaged mitochondria [391, 392]. UFD1L also participates in ER-associated degradation, spindle disassembly, DNA-damage response, cell-cycle control, telomerase length regulation, and ribosome-associated degradation [393–397]. *UFD1L* functional attenuation in chick cardiac neural crest increases conotruncal septation defects [398]. *Ufd1l* is expressed in pharyngeal arches, palatal precursors, and developing ears, heart, and brain (Fig. 1c, d) [188, 399]. *Ufd1l*^−/−^ embryos die before organogenesis [398]. In human, *UFD1L* expression is reduced in bicuspid aortic valve, a common cardiac defect, further supporting a role in proper heart formation [400]. *Drosophila ufd1* is implicated in neuronal function and maintenance [401]. Its role in mouse and human brain function is less clear. In the *LgDel* brain, *Ufd1L* transcripts decline by approximately 50%, but Ufd1L protein is at WT levels [311]. *UFD1L* polymorphisms are associated with increased corpus callosum volumes and have been linked to cognitive deficits independent of 22q11.2 deletion [402, 403].

*COMT Catechol-O-Methyltransferase* (**A to B**) is one of several enzymes that degrade catecholamines, including dopamine, epinephrine, and norepinephrine [404]. COMT membrane-bound and soluble isoforms are predominately expressed in brain and peripheral tissues, respectively [404, 405]. *Comt* is expressed in several brain regions (Fig. 1c, d). Mutant mice lacking *Comt* show impaired emotional reactivity and cognition as well as increased aggressive behaviors [406, 407]. COMT polymorphisms are linked independently to SCZ, bipolar disorder, obsessive-compulsive disorder, anorexia, and ADHD [20, 408–410]—many are also 22q11DS behavioral phenotypes. This correlation suggests a potential role for COMT in regulating dopamine levels, which are disrupted in most of these disorders [411]. COMT association to SCZ, however, was challenged in a recent genome-wide association study [327].

*TSSK2 Testis Specific Serine Kinase 2* (a.k.a. *STK22b*; **A to B**) encodes a serine/threonine kinase involved in spermatogenesis [412]. In mouse, the *Stk22b* orthologue (*STK22a* is a pseudogene) is not expressed at 22q11.2 phenotypic sites (heart, face, limbs, thymus, brain), nor detected in the maturing or adult brain [58]. Recently, one patient with 22q11DS has been reported to have azoospermia, which could reflect *TSSK2* deficiency [413]*.*

*TRMT2a tRNA Methyltransferase 2 Homolog A* (a.k.a. *HTF9C*; **A to B**) is a tRNA methyltransferase with homology to a yeast *scTrm2* DNA repair enzyme [414]. *TRMT2a* is one of 34 human tRNA methyltransferases that target approximately 600 tRNA variants encoded by the human genome [415] to modulate tRNA stability, translational efficiency, and protein production. TRMT2A may contribute to 22q11DS phenotypes; however, genomic diversity of tRNA methyltransferases and their tRNA targets raises the possibility of functional redundancy and compensation. *Trmt2a* is expressed in the heart, pharyngeal arches, and brain (Fig. 1c) [58]. *TRMT2a* and its murine orthologue share a bidirectional promoter with *Ranbp1*, an established 22q11.2 contributor gene (see above); however, *Trmt2a* is expressed at WT levels in *Ranbp1*^−/−^ mice [170]. Promoter polymorphisms in the *TRMT2A/RANBP1* locus, however, could yield additional dosage-dependent changes in 22q11DS.

### Perspective: additional genes with unique functions

All “sui generis” 22q11.2 genes are in the **A to B** region, implicating each as a potential contributor to key phenotypes. Of the four, *TSSK2* seems least likely to contribute to neural, cardiovascular, or craniofacial development. Similarly, *UFD1L*, if translationally dosage compensated in humans as in mice, is also less likely. *DGCR6* merits further consideration following additional mechanistic studies. *COMT* remains a prime suspect based upon its contributions to aminergic neural signaling and potential association with behavioral disorders.

## Conclusion

### Single suspects and accomplices from the 22q11.2 gene line-up

**Fig. 5:**
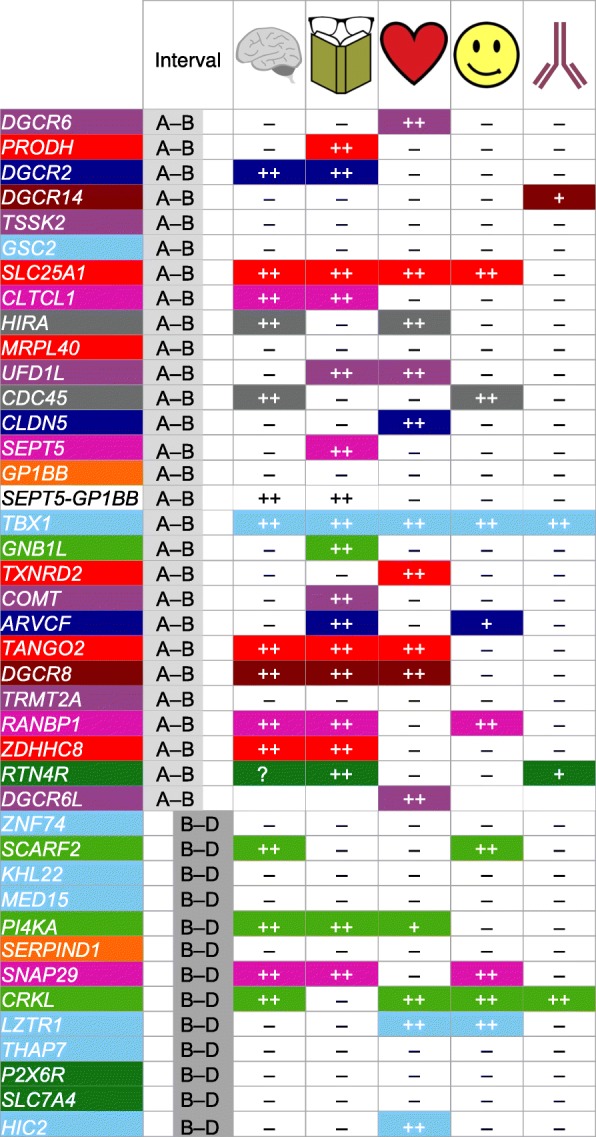
22q11.2 genomic matrix for heart, neural, craniofacial and immune phenotypes. The table represents proximal-distal alignment of forty-one characterized protein-coding genes in 22q11.2 deleted region and their apparent functions in neural, cognitive, cardiovascular, craniofacial, and immune development. The presence of a non-consensus polyA signal in *SEPT5* gene results in read-through transcription into the downstream neighboring gene resulting in *SEPT5*-*GP1BB* transcript “Entrez Gene: SEPT5”. “++”: if there is evidence from human and/or mouse, “+” if the evidence is from in vitro data or an investigation in lower vertebrates, “−” if there is no evidence the gene affects this particular function, and “?” if the data is inconclusive

**Fig. 6:**
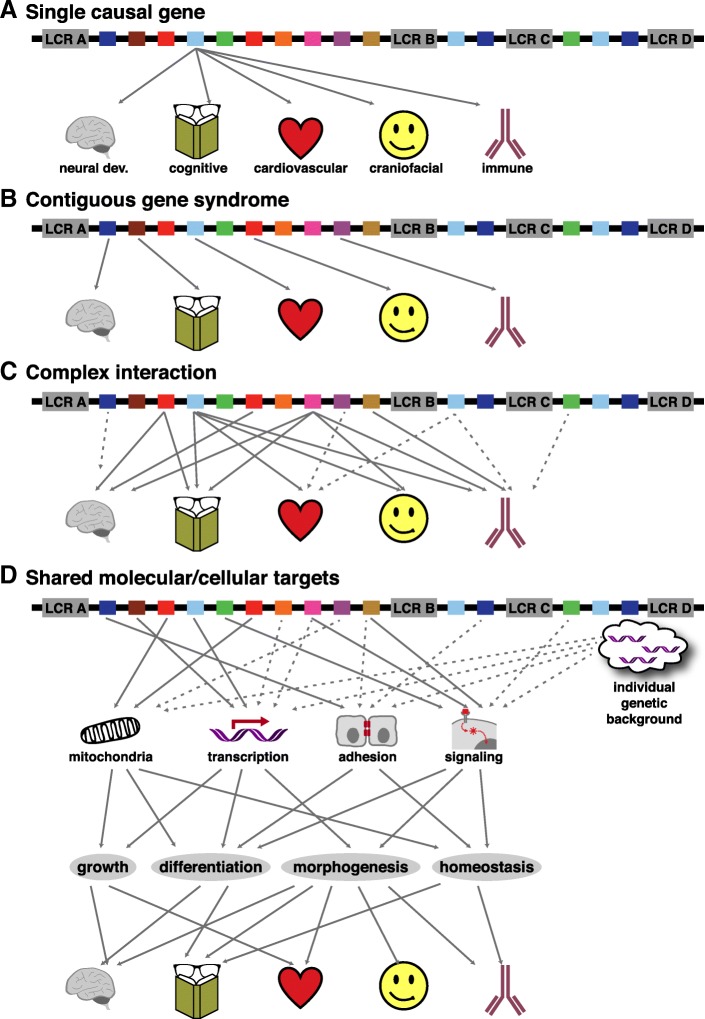
Possible mechanism for 22q11.2 gene interaction. **a** Single causal gene; a single gene is responsible for all core 22q11DS phenotypes. **b** Contiguous genes; a single gene independently leads to each key 22q11DS phenotype. **c** Gene interactions; a combination of multiple genes are cooperatively responsible for each core 22q11DS phenotype. **d** Common functional targets; multiple genes conspire to disrupt key shared mechanisms, compromising key aspects of development or homeostasis at multiple 22q11DS phenotypic sites. “Individual genetic background” indicates contributions from allelic variants of genes outside the deletion region. The arrows are hypothetical illustrations of how multiple mechanisms could interact. Dotted arrows indicate weaker interactions

Over the last decade, data addressing developmental roles of several 22q11.2 genes have increased dramatically based on extrapolation of full loss-of-function phenotypes caused by deletion of single 22q11.2 orthologues in mouse and other model species. Nevertheless, the relationships between altered dosage of 22q11.2 genes—rather than homozygous loss of function—and the many and variable phenotypes in individuals with 22q11DS remain unclear. The spectrum of speculations regarding which gene “explains” 22q11.2 phenotypes is as broad as the 22q11DS phenotypic spectrum itself. Proposed explanations range from single gene haploinsufficiency accounting for all essential 22q11DS phenotypes, to more nuanced contiguous gene or gene-gene interactions that lead to subsets of clinically significant features. Several “genes of interest” have been identified; however, the potential contribution of several others—both confirmed coding loci as well as unannotated open reading frames with uncertain protein coding function as well as microRNAs—have not been thoroughly considered. We revisited the “line-up” of deleted 22q11.2 genes—focusing on those that encode identified proteins detected in at least one tissue in humans or model organisms—in the 3 Mb, typically deleted, **A to D** region. Our assessment of primary cellular functions and mutant dysfunction of all known protein coding 22q11.2 genes allows us to address two unsolved questions essential for understanding 22q11DS pathogenic mechanisms: (1) which phenotypes reflect true haploinsufficiency for **A to B** or **A to D** genes? (2) What causes phenotypic variability in individuals with distinct or even identical deletions? This 22q11.2 gene “line-up,” reflecting insight from human and model system genetic analysis as well as parallel cell biological and molecular assessments establishes a genetic interaction matrix (Fig. 5) that generates multiple outcomes with differing severity (Fig. 6). Understanding the detailed nature of this matrix, we suggest, will yield better therapeutic options for clinical challenges faced by individuals with 22q11DS. Additional investigation is necessary to complete the suspect list and decipher interactions that modify or diversify key 22q11DS features. Future studies targeting gene “cabals” in animal models should elucidate additional gene-gene interactions within the phenotypic matrix defined by **A to B** and **B to D** deletions (Figs. 5 and 6). Nevertheless, this matrix alone cannot reliably predict combinations of dosage-dependent phenotypes due to 22q11.2 deletions. Indeed, these predictions might be generated by combining the 22q11.2 interaction matrix with data on individual polymorphisms beyond 22q11.2 genes as well as fetal and post-natal environmental exposures [73, 264].

### Candidate genes

“Which gene?” remains a central question for CNV-associated developmental disorders. If 22q11DS diagnostic assessment and therapies are to be targeted to pathogenic processes and delivered at appropriate times to maximize benefits in 22q11DS and other CNV-associated syndromes, it is imperative to identify culpable genes, characterize their obligate functions, and relate those data to disease-causing mechanisms and individual variability. Based upon evidence gathered by multiple investigators, it is unlikely that haploinsufficiency of a single 22q11.2 gene accounts for the full range of 22q11DS-related phenotypes. Nevertheless, our “line-up” indicates new promising leads on mechanistic interactions between suspects that disrupt development or homeostasis in 22q11DS. At least three possibilities—and one red herring—emerge from our functional genomic synthesis of minimally and typically deleted 22q11.2 genes.

#### The red herring

A *single* gene explains core 22q11DS phenotypes (Fig. 6a). Beyond doubt, several **A to B** as well as **B to D** genes, due to loss-of-function mutations or diminished dosage, contribute substantially, yet variably, to 22q11DS-related phenotypes. Nevertheless, the conclusion that any single gene “acts alone” to cause even one, let alone the full spectrum of 22q11DS phenotypes, without being influenced by diminished dosage of others, is no longer tenable based on judicious assessment of the evidence summarized here. What, then, more viable leads emerge from our thorough investigation of individual 22q11.2 genes and the primary cellular and developmental function of the proteins they encode?

#### First

22q11DS is a classic “contiguous gene syndrome” (Fig. 6b) in which diminished dosage of single **A to B** genes independently leads to each key 22q11.2 phenotype: one for the brain, one for the heart, one for the face, and one for the thymus. In this scenario, **B to D** genes may modulate each **A to B** gene’s singular influence, but do not account for the core phenotypic change. Differing phenotypic frequencies in single gene heterozygous mutants versus broader deletion (see Fig. 3), or stochastic interactions between **A to B** and **B to D** genes and signals like sonic hedgehog (Shh), RA, or FGF [73, 74, 217, 416] suggest this is unlikely.

#### Second

Gene subsets form multigenic “syndicates” to disrupt critical developmental mechanisms or homeostatic function at *specific* phenotypic sites (Fig. 6c). For example, diminished dosage of *Crkl* **(B to D)**, combined with that of *Tbx1* (**A to B**) and a few other **A to B** genes may be responsible for many of the core cardiovascular anomalies as well as their variability [417]. Other distinct groups of 22q11 genes might similarly impact the brain, face, or thymus. There is considerable evidence for these sorts of selective, phenotype-specific interactions, at least in the heart, and most of this evidence has been reviewed here.

#### Third

A broader “interactome” is impacted by dosage-related changes in which proteins of similar cellular function encoded by multiple **A to B** genes, modulated by **B to D** genes, act in concert to disrupt essential cellular mechanisms at several phenotypic sites, rather than to elicit one single phenotype. These interactions may be due to sequential disruption of cell biological mechanisms leading to a threshold of developmental or homeostatic dysfunction at multiple phenotypic sites, or more direct, but locally distinct, interactions among functionally related 22q11.2 proteins in different organs and tissues. Such fundamental 22q11.2 dosage-dependent interactions may then be further modified by an individual’s genomic background independent of 22q11.2 deletion (Fig. 6d). In this scenario, 22q11.2 genes with similar function, particularly mitochondrial, transcription factor, adhesion molecules, or signaling factors—some only in the **A to B** interval, others from **B to D**—disrupt cellular mechanisms common to development and/or maintenance of heart, face, immune, skeletal, and brain integrity.

Additional evidence supports this “interactome” hypothesis as a viable solution to the yet unsolved mystery of 22q11.2 dosage-dependent phenotypic change and variability. Within the 22q11.2 deleted region, ARVCF and RANBP1 (**A to B)** as well as CRKL and SNAP29 (**B to D**) are predicted to interact with cadherin 1 (CDH1). Such interactions, influenced by stoichiometric change in proteins encoded by 22q11.2 genes, and modulated by polymorphisms in CDH1 in this example, or additional loci for other subsets of functionally related 22q11.2 proteins, could generally alter adhesion-dependent morphogenesis at all of 22q11DS phenotypic sites. CRKL (**B to D**), implicated in *TBX1*-dependent phenotypic changes, also interacts with phosphatase and tensin homolog (PTEN), a lipid phosphatase in the PI3K-AKT signaling pathway, involved in cell growth, proliferation, survival, and migration [418]. In addition, CRKL as well as DGCR8 interact with proteins involved in mRNA maturation and degradation, further supporting convergence on distinct cellular mechanisms that could compromise multiple phenotypic sites as more plausible mechanism for 22q11DS pathogenesis. The global instability of transcriptional regulation and its stochastic effects on 22q11 genes, perhaps effectively lowering their expression levels below a “loss of function” threshold on a cell-by-cell basis might broadly compromise multiple steps of development and maintenance of the heart, thymus, limbs, face, and brain. Analysis of genomic, transcriptomic, and proteomic databases can generate additional hypotheses of this sort of cellular mechanistic convergence. These hypotheses can then be tested genetically in the most appropriate model system among the several described here, or analyzed in cell lines, or in induced pluripotent stem cells (iPSCs) from several individuals with 22q11.2 deletions to further assess variability. Critically, one would predict that experiments in which key modulators of the cell biological mechanisms upon which subsets of 22q11.2 genes converge (see Fig. 6) were manipulated genetically or pharmacologically would provide further confirmation of the third scenario.

Our gene dosage-based/convergence-upon-cellular-mechanism hypothesis for 22q11DS is generally consistent with recent genomic analyses in non-syndromic individuals diagnosed with two behavioral disorders that are also significantly associated with 22q11DS: ASD and SCZ. There may be similar convergence on essential cellular mechanisms in ASD: 65 risk genes have been identified as chromatin regulators or establishment and maintenance of synaptic stability [21]. Multiple genes that encode cell adhesion molecules, tight junction proteins, and regulators of vesicle trafficking—all involved in critical processes for neural circuit development and neuronal signaling—have been implicated in SCZ susceptibility [22, 23]. Apparently, multigenic origins of complex brain disorders like ASD and SCZ may reflect a combination of single gene variants that independently are benign, but as a group, target common cellular mechanisms leading to dysfunction. Viewing these variants as independent actors, rather than interactors in a network, might result in incomplete explanations of pathology in clinically defined diseases like ASD, SCZ, or in multi-gene CNV syndromes like 22q11DS. New efforts to evaluate concerted actions of multiple genes on focal mechanisms for brain development and function are needed to advance our understanding of neurodevelopmental disorders generally. Our newly assembled gene 22q11.2 “line-up,” from LCR A to LCR D, allows us to view “suspect” genes anew and formulate new investigative strategies for 22q11DS, perhaps providing a template for understanding a wider range of multigenic or CNV-associated syndromes.

## Additional file

Additional file 1: **Fig. S1.** Human 22q11.2 region gene entries. There are 154 GenBank entries in the 3 Mb deleted region of hChr22 between LCR A and LCR D at position q11.2. This includes 56 predicted protein-coding genes (white), 7 microRNAs (yellow), 38 non-coding RNAs (blue) and 53 pseudogenes (green). The gray areas represent predicted protein coding loci within the LCR regions. (PDF 66 kb)

## Abbreviations

22q11DS: 22q11.2 deletion syndrome
Ac-LDL: Acetylated low-density lipoprotein
ADHD: Attention deficit hyperactivity disorder
ASD: Autistic spectrum disorders
BTB: Bric-a-brac-Tramtrack-Broad complex
CAT: Cationic amino acid transporters
CNs: Cranial nerves
CNV: Copy number variation
CP: Cortical plate
CTAF: Conotruncal anomaly face syndrome
Dmd1: Dystrophin
Dpp: Decapentaplegic
EGF: Epidermal growth factor
ER: Endoplasmic reticulum
FGF: Fibroblast growth factor
GEFs: Guanine-nucleotide exchange factors
GPI: Glycosylphosphatidylinositol
hChr22: Human chromosome 22
IPN: Interpeduncular nucleus
iPSCs: Induced pluripotent stem cells
IZ: Intermediate zone
Iα/β: Importins
KRAB: Kruppel-associated box
LCR: Low copy number repeats
NES: Nuclear export signal
NLS: Nuclear localization signal
PI3: Phosphoinositide 3-kinase
PLC: Phospholipase C
PPI: Pre-pulse inhibition
PtdIns4*P*: Phosphatidylinositol 4-phosphate
PTEN: Phosphatase and tensin homolog
RA: Retinoic acid
SCZ: Schizophrenia
snoRNA: Small nucleolar RNAs
SP: Subplate
Utrn: Utrophin
VCFS: Velo-cardio-facial syndrome
VZ: Ventricular zone
Xpo1: Exportin

## Authors’ information

Zahra Motahari, Ph.D. Post-doctoral Associated, Department of Anatomy and Cell Biology, The George Washington University School of Medicine and Health Sciences, Washington DC, 20037 motahariz@gwu.edu

Sally A. Moody, Ph.D. Professor and Chair, Department of Anatomy and Cell Biology, The George Washington University School of Medicine and Health Sciences, Washington DC, 20037.

samoody@gwu.edu

Thomas M. Maynard Ph.D., Associate Research Professor, Department of Anatomy and Cell Biology, The George Washington University School of Medicine and Health Sciences, Washington DC, 20037.

maynard@gwu.edu

Anthony-Samuel LaMantia, Ph.D. Lieberman Professor of Neuroscience, Director, GW Institute for Neuroscience, Department of Anatomy and Cell Biology, The George Washington University School of Medicine and Health Sciences, Washington DC, 20037.

lamantia@gwu.edu
