# Supplementary material for: In the line-up: deleted genes associated with DiGeorge/22q11.2 deletion syndrome: are they all suspects?
Source: J Neurodev Disord. 2019 Jun 7;11:7. doi: 10.1186/s11689-019-9267-z (PMC6554986; doi:10.1186/s11689-019-9267-z)
Supplement: Supplementary file 1 — Fig. S1. Human 22q11.2 region gene entries. There are 154 GenBank entries in the 3 Mb deleted region of hChr22 between LCR A and LCR D at position q11.2. This includes 56 predicted protein-coding genes (white), 7 microRNAs (yellow), 38 non-coding RNAs (blue) and 53 pseudogenes (green). The gray areas represent predicted protein coding loci within the LCR regions. (PDF 66 kb) [file 11689_2019_9267_MOESM1_ESM.pdf]

Human 22q11.2 region gene entries   Genes in Region: **154**   (1/2)

	start	stop	Symbol	Strand	Name	Type
LCR A	18178026	18205863	LOC100996415	-	uncharacterized LOC100996415	ncRNA
	18209155	18211924	LOC105372858	+	uncharacterized LOC105372858	ncRNA
	18220562	18224162	LOC101060852	+	protein phosphatase 1 regulatory subunit 26-like	psedo
	18233149	18235228	LOC105379518	+	uncharacterized LOC105379518	ncRNA
	18340163	18346065	GGTLC5P	+	gamma-glutamyltransferase light chain 5 pseudogene	pseudo
	18349938	18361189	LOC105372942	+	uncharacterized LOC105372942	ncRNA
	18361830	18386526	LINC01660	-	long intergenic non-protein coding RNA 1660	ncRNA
	18401898	18404263	PPP1R26P3	-	protein phosphatase 1 regulatory subunit 26 pseudogene 3	pseudo
	18412708	18415486	LOC107987325	-	uncharacterized LOC107987325	ncRNA
	18487127	18500594	FAM230A	+	family with sequence similarity 230 member A	ncRNA
	18516338	18518161	GGTLC3	-	gamma-glutamyltransferase light chain family member 3	protein
	18527054	18527702	LOC100996401	+	transmembrane protein 191A	protein
	18527802	18531920	TMEM191B	+	transmembrane protein 191B	protein
	18533864	18548828	PI4KAP1	-	phosphatidylinositol 4-kinase alpha pseudogene 1	pseudo
	18605865	18606102	RN7SKP131	-	RNA, 7SK small nuclear pseudogene 131	pseudo
	18606124	18611919	RIMBP3	-	RIMS binding protein 3	protein
	18623339	18625227	SUSD2P2	-	sushi domain containing 2 pseudogene 2	pseudo
	18634541	18635687	LOC105372871	+	uncharacterized LOC105372871	ncRNA
	18635987	18637497	CA15P2	-	carbonic anhydrase 15 pseudogene 2	pseudo
	18649828	18653812	PPP1R26P2	-	protein phosphatase 1 regulatory subunit 26 pseudogene 2	pseudo
	18715620	18719643	PPP1R26P4	+	protein phosphatase 1 regulatory subunit 26 pseudogene 4	pseudo
	18733914	18757894	LINC01662	+	long intergenic non-protein coding RNA 1662	ncRNA
	18758535	18769783	LOC105377182	-	uncharacterized LOC105377182	ncRNA
	18773689	18791961	GGT3P	-	gamma-glutamyltransferase 3 pseudogene	pseudo
	18833419	18835626	E2F6P1	+	E2F transcription factor 6 pseudogene 1	pseudo
	18846449	18848852	LOC101929738	+	putative POM121-like protein 1	pseudo
	18850118	18861064	LOC102725072	+	Putative uncharacterized protein DKFZp434K191	pseudo
	18852284	18858640	BCRP7	-	breakpoint cluster region pseudogene 7	pseudo
	18865043	18894431	LINC01663	-	long intergenic non-protein coding RNA 1663	ncRNA
	18906223	18912088	DGCR6	+	DiGeorge syndrome critical region 6	protein
	18912774	18936553	PRODH	-	proline dehydrogenase 1	protein
	18970498	19031242	DGCR5	+	DiGeorge syndrome critical region gene 5	ncRNA
	19017834	19020248	DGCR9	+	DiGeorge syndrome critical region gene 9	ncRNA
	19022624	19023550	DGCR10	+	DiGeorge syndrome critical region gene 10	ncRNA
	19036282	19122454	DGCR2	-	DiGeorge syndrome critical region gene 2	protein
	19046162	19048375	DGCR11	-	DiGeorge syndrome critical region gene 11	ncRNA
	19055841	19056187	LOC100129262	+	ribosomal protein L28 pseudogene	pseudo
	19124874	19125887	TSSK1A	+	testis specific serine kinase 1A, pseudogene	pseudo
	19130279	19144726	ESS2	-	ess-2 splicing factor homolog (DGCR14)	protein
	19130808	19132623	TSSK2	+	testis specific serine kinase 2	protein
	19148991	19150283	GSC2	-	goosecoid homeobox 2	protein
	19171706	19172832	LINC01311	+	long intergenic non-protein coding RNA 1311	ncRNA
	19175575	19178863	SLC25A1	-	solute carrier family 25, member 1	protein
	19179473	19291716	CLTCL1	-	clathrin heavy chain like 1	protein
	19238443	19238739	RPL34P35	-	ribosomal protein L34 pseudogene 35	pseudo
	19253121	19253469	DVL1P1	-	dishevelled segment polarity protein 1 pseudogene 1	pseudo
	19257518	19258861	KRT18P62	+	keratin 18 pseudogene 62	pseudo
	19291796	19351456	LOC105372859	+	uncharacterized LOC105372859	ncRNA
	19330701	19431696	HIRA	-	histone cell cycle regulator	protein
	19365765	19366044	RN7SL168P	+	RNA, 7SL, cytoplasmic 168, pseudogene	pseudo
	19431902	19436078	MRPL40	+	mitochondrial ribosomal protein L40	protein
	19440886	19448232	C22orf39	-	chromosome 22 open reading frame 39	protein
	19447568	19454605	LOC105372860	+	uncharacterized LOC105372860	ncRNA
	19449941	19479215	UFD1	-	ubiquitin recognition factor in ER associated degradation 1	protein
	19479278	19520612	CDC45	+	cell division cycle 45	protein
	19523024	19527545	CLDN5	-	claudin 5	protein
	19566130	19566839	LINC00895	-	long intergenic non-protein coding RNA 895	ncRNA
	19630429	19632169	LOC100129254	-	chromosome 3 open reading frame 38 pseudogene	pseudo
	19659258	19659814	LOC100420103	-	uncharacterized LOC100420103	pseudo
	19714464	19723322	5-Sep	+	septin 5	protein
	19717220	19724774	SEPT5-GP1BB	+	SEPT5-GP1BB readthrough	protein
	19723543	19724774	GP1BB	+	glycoprotein Ib platelet beta subunit	protein
	19727721	19738960	LOC105372861	+	uncharacterized LOC105372861	ncRNA
	19756703	19783593	TBX1	+	T-box 1	protein
	19788411	19854939	GNB1L	-	G protein subunit beta 1 like	protein
	19792275	19793153	RPL7AP70	+	ribosomal protein L7a pseudogene 70	pseudo
	19846138	19854848	RTL10	-	retrotransposon Gag like 10	pseudo
	19875518	19941992	TXNRD2	-	thioredoxin reductase 2	protein
	19887289	19887970	RPL8P5	+	ribosomal protein L8 pseudogene 5	pseudo
	19941740	19969975	COMT	+	catechol-O-methyltransferase isoform	protein
	19963753	19963834	MIR4761	+	microRNA 4761	microRNA
	19966491	20016802	ARVCF	-	ARVCF, delta catenin family member	protein
	20017000	20067164	TANGO2	+	transport and golgi organization 2 homolog	protein
	20033139	20033220	MIR185	+	microRNA 185	microRNA
	20080232	20111877	DGCR8	+	DGCR8, microprocessor complex subunit	protein
	20085746	20085833	MIR3618	+	microRNA 3618	microRNA
	20086058	20086142	MIR1306	+	microRNA 1306	microRNA
	20111866	20117305	TRMT2A	-	tRNA methyltransferase 2 homolog A	protein

Human 22q11.2 region gene entries

	start	stop	Symbol	Strand	Name	Type
	20114686	20114751	MIR6816	-	microRNA 6816	microRNA
	20115938	20127357	RANBP1	+	RAN binding protein 1	protein
	20131841	20148007	ZDHHC8	+	zinc finger DHHC-type containing 8	protein
	20148113	20151828	CCDC188	-	coiled-coil domain containing 188	protein
	20198730	20204918	LOC284865	-	uncharacterized LOC284865	ncRNA
	20206037	20213800	LOC105372862	-	uncharacterized LOC105372862	ncRNA
	20206332	20208537	LINC00896	+	long intergenic non-protein coding RNA 896	ncRNA
	20226945	20235971	LOC105372863	+	uncharacterized LOC105372863	ncRNA
	20241415	20268293	RTN4R	-	reticulon 4 receptor	protein
	20244793	20253854	LOC105372864	+	uncharacterized LOC105372864	ncRNA
	20249134	20249211	MIR1286	-	microRNA 1286	microRNA
LCR B	20297310	20313590	LOC440792	+	proline dehydrogenase 1 pseudogene	pseudo
	20314238	20320105	DGCR6L	-	OTTHUMP00000197880	protein
	20340997	20352494	LOC101927859	+	uncharacterized LOC101927859	ncRNA
	20363621	20390758	USP41	-	OTTHUMP00000199828	protein
	20394115	20408463	ZNF74	+	zinc finger protein 74	protein
	20416798	20416899	RNU6-225P	-	RNA, U6 small nuclear 225, pseudogene	pseudo
	20424584	20437859	SCARF2	-	hCG_40491	protein
	20437970	20443203	LOC107985588	+	uncharacterized LOC107985588	ncRNA
	20441519	20496249	KLHL22	-	kelch like family member 22	protein
	20449901	20453240	LOC100420177	+	kelch like family member 12 pseudogene	pseudo
	20475194	20475311	RNY1P9	-	RNA, Ro-associated Y1 pseudogene 9	pseudo
	20481935	20482213	RN7SL812P	-	RNA, 7SL, cytoplasmic 812, pseudogene	pseudo
	20482748	20484155	KRT18P5	-	keratin 18 pseudogene 5	pseudo
	20495948	20500878	LOC101928824	+	uncharacterized LOC101928824	ncRNA
	20507542	20587632	MED15	+	mediator complex subunit 15	protein
	20589576	20592352	CCDC74BP1	-	coiled-coil domain containing 74B pseudogene 1	pseudo
	20592959	20603267	LOC107985587	-	uncharacterized LOC107985587	ncRNA
LCR C	20604895	20626185	SMPD4P1	-	sphingomyelin phosphodiesterase 4 pseudogene 1	pseudo
	20632590	20632826	IGLL4P	+	immunoglobulin lambda like polypeptide 4, pseudogene	pseudo
	20639072	20650483	LOC100421121	-	small G protein signaling modulator 1 pseudogene	pseudo
	20652644	20654727	SLC9A3P2	+	solute carrier family 9 member 3 pseudogene 2	pseudo
	20667597	20671339	ABHD17AP4	-	abhydrolase domain containing 17A pseudogene P4	pseudo
	20689555	20691721	POM121L4P	+	POM121 transmembrane nucleoporin like 4, pseudogene	pseudo
	20695438	20698959	BCRP5	-	breakpoint cluster region pseudogene 5	pseudo
	20696566	20698250	LOC107985584	+	uncharacterized LOC107985584	ncRNA
	20701114	20704603	TMEM191A	+	transmembrane protein 191A (pseudogene)	pseudo
	20707691	20858812	PI4KA	-	phosphatidylinositol 4-kinase, catalytic, alpha	protein
	20774095	20787720	SERPIND1	+	serpin family D member 1	protein
	20859004	20891214	SNAP29	+	synaptosome associated protein 29	protein
	20917398	20953749	CRKL	+	CRK like proto-oncogene, adaptor protein	protein
	20957092	20964680	LINC01637	+	long intergenic non-protein coding RNA 1637	ncRNA
	20965130	20981360	AIFM3	+	apoptosis inducing factor, mitochondria associated 3	protein
	20982269	20999037	LZTR1	+	leucine zipper like transcription regulator 1	protein
	20999772	21002115	THAP7	-	THAP domain containing 7	protein
	21001922	21010374	THAP7-AS1	+	THAP7 antisense RNA 1	ncRNA
	21008203	21014287	TUBA3FP	-	tubulin alpha 3f pseudogene	pseudo
	21009699	21028013	P2RX6	+	purinergic receptor P2X 6	protein
	21028718	21032558	SLC7A4	-	OTTHUMP00000198675	protein
	21034176	21034272	MIR649	-	microRNA 649	microRNA
LCR D	21042392	21044249	P2RX6P	-	purinergic receptor P2X 6 pseudogene	pseudo
	21045960	21064168	LRRC74B	+	leucine rich repeat containing 74B	protein
	21065467	21070173	TUBA3GP	+	tubulin alpha 3g pseudogene	pseudo
	21103016	21122286	BCRP2	+	breakpoint cluster region pseudogene 2	pseudo
	21112788	21128092	POM121L7P	-	POM121 transmembrane nucleoporin like 7 pseudogene	pseudo
	21140441	21142648	E2F6P2	-	E2F transcription factor 6 pseudogene 2	pseudo
	21166903	21192156	FAM230B	+	family with sequence similarity 230 member B	ncRNA
	21192790	21204020	LOC105372935	-	uncharacterized LOC105372935	ncRNA
	21207972	21282982	GGT2	-	gamma-glutamyltransferase 2	protein
	21267884	21270091	E2F6P3	+	E2F transcription factor 6 pseudogene 3	pseudo
	21282425	21297730	POM121L8P	+	POM121 transmembrane nucleoporin like 8, pseudogene	pseudo
	21288229	21294589	BCRP6	-	breakpoint cluster region pseudogene 6	pseudo
	21301260	21326636	LOC100996335	-	uncharacterized LOC100996335	ncRNA
	21341401	21343767	PPP1R26P5	+	protein phosphatase 1 regulatory subunit 26 pseudogene 5	pseudo
	21354242	21357968	LINC01651	+	long intergenic non-protein coding RNA 1651	ncRNA
	21359596	21360702	LOC105377190	-	uncharacterized LOC105377190	ncRAN
	21370061	21371949	SUSD2P1	+	sushi domain containing 2 pseudogene 1	pseudo
	21383751	21389169	RIMBP3B	+	RIMS binding protein 3B	protein
	21389191	21389428	RN7SKP63	+	RNA, 7SK small nuclear pseudogene 63	pseudo
	21417402	21451463	HIC2	+	HIC ZBTB transcriptional repressor 2	protein
	21419761	21422416	LOC105377191	-	uncharacterized LOC105377191	ncRNA
	21467170	21469935	TMEM191C	+	transmembrane protein 191C	protein
	21472998	21517491	PI4KAP2	-	phosphatidylinositol 4-kinase alpha pseudogene 2	pseudo
	21545407	21545644	RN7SKP221	-	RNA, 7SK small nuclear pseudogene 221	pseudo
	21545666	21551461	RIMBP3C	-	RIMS binding protein 3C	protein
	21549447	21624034	UBE2L3	+	ubiquitin conjugating enzyme E2 L3	protein
